# Supplementary material for: Unique properties of a subset of human pluripotent stem cells with high capacity for self-renewal
Source: Nat Commun. 2020 May 15;11:2420. doi: 10.1038/s41467-020-16214-8 (PMC7229198; doi:10.1038/s41467-020-16214-8)
Supplement: Supplementary file 1 — Supplementary Information [file 41467_2020_16214_MOESM1_ESM.pdf]

Supplementary Material Contents

1. Supplementary Figure 1: Analysis of cell cycle distribution of hPSC cell subpopulations using the FUCCI reporter system.
2. Supplementary Figure 2. Analysis of metabolomics data for the GCTM-2$_{high}$CD9$_{high}$EPCAM$_{high}$ (HHH) and remaining (GEN) cells.
3. Supplementary Figure 3. Expression of *TET1*, *DNMT3A*, and *DNMT3B* in subpopulations of hPSC.
4. Supplementary Figure 4. ATAC-seq library quality control.
5. Supplementary Figure 5. Regions of increased DNA accessibility in GCTM-2$_{high}$CD9$_{high}$EPCAM$_{high}$ (high) population are enriched for stem cell-specific chromatin sites.
6. Supplementary Figure 6. Heatmaps showing distribution of differentially expressed genes in GCTM-2$_{high}$CD9$_{high}$EPCAM$_{high}$ (a) and unsorted cells (b, general population, GEN) across the cortical neural differentiation time course described in Cortecon (29).
7. Supplementary Figure 7. Data from RNA microarray analyses in [1] showing expression in stem cell subpopulations of genes upregulated in the General population in this work.
8. Supplementary Figure 8. Distribution of gene expression in processed single cell RNA-seq for human and non-human primate datasets.
9. Supplementary Figure 9. Z-scores of ranked expression per cell.
10. Supplementary Figure 10. Single cell gene expression analysis for pluripotency associated transcription factors in subsets of hPSC. a, principal component analysis of cell subsets. b, POU5F1. c, SOX2. d, NANOG. e, ZFP42. f, TFCP2L1. G, POU3F1.
11. Supplementary Figure 11. Principal component analysis of single cell RNA-seq cynomolgus embryo data from [30]; single embryo cells classified according to [30].
12. Supplementary Figure 12. Exemplary flow cytometry gating strategies for this study. a, strategy for isolation of GCTM-2$_{high}$CD9$_{high}$EPCAM$_{high}$ cells (Figures 2-9). b, strategy for isolation of GCTM-2$_{high}$CD9$_{high}$ and GCTM-2$_{low}$CD9$_{low}$ cells and determination of mitochondrial membrane potential with JC1 or TMRM (Figure 3). c, strategy for enumeration of ITGA6^{+}/EPCAM^{+} cells (Figure 2).
13. Supplementary Movie 1: Fate of aggregates of GCTM-2$_{high}$CD9$_{high}$ cells for 1-24 hours post-plating
14. Supplementary Movie 2: Fate of aggregates of GCTM-2$_{mid}$CD9$_{mid}$ cells for 1-24 hours post-plating.
15. Supplementary Table 1. LC-MS analysis showing metabolites that differed significantly between GCTM-2$_{high}$CD9$_{high}$EPCAM$_{high}$ subpopulation and remaining cells (general population).
16. Supplementary Table 2. GC-MS analysis showing metabolites that differed significantly between GCTM-2$_{high}$CD9$_{high}$EPCAM$_{high}$ subpopulation and unsorted cells (general population).

17. Supplementary Data 1. Differential chromatin accessible peaks between populations.
18. Supplementary Data 2. Results from locus overlap enrichment analysis (LOLA) for chromatin accessible peaks from GCTM-2_{high}CD9_{high}EPCAM$_{high}$ and GCTM-2_{mid}CD9_{mid} populations.
19. Supplementary Data 3. RNA-seq analysis of differential gene expression in GCTM-2_{high}CD9_{high}EPCAM$_{high}$ subpopulation and unsorted cells (general population).
20. Supplementary Table 3. Pathway analysis of differentially expressed genes in GCTM-2_{high}CD9_{high}EPCAM$_{high}$ subpopulation and unsorted cells (general population) identified by RNA-seq.
21. Supplementary Table 4. Stage-specific differentially expressed genes.
22. Supplementary Table 5. Biological replicate data for assay in Figure 1e showing colony formation of single cell and cell aggregates from GCTM-2_{high}CD9_{high} and GCTM-2_{mid}CD9_{mid} subpopulations.
23. Supplementary Table 6. Biological replicate data for cell cycle studies using Fucci reporter system shown in Supplementary Figure 1.

Supplementary Figure 1

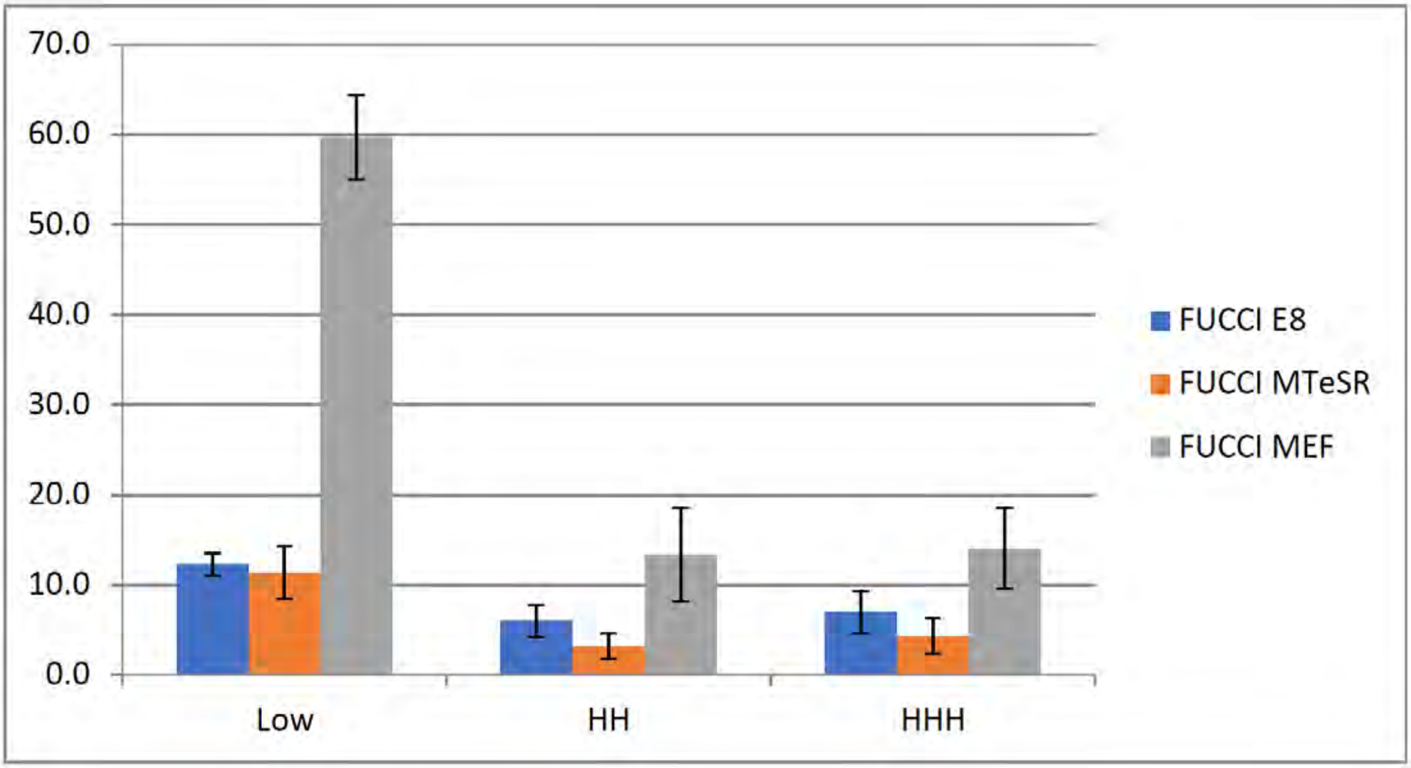

Supplementary Figure 1. Analysis of cell cycle distribution of hPSC cell subpopulations using the FUCCI reporter system. The graph shows the proportion of cells in G0/G1 for the GCTM-2^{low}CD9^{low} (LOW), GCMT-2^{high}CD9^{high} (HH), and GCTM-2^{high}CD9^{high}EPCAM^{high} (HHH) subpopulations for WA09 cultures grown in E8 defined medium (FUCCI E8), MTeSR defined medium (FUCCI mTeSR), or in serum-containing medium with mouse embryo fibroblast feeder cell support (FUCCI MEF). Values are means and error bars represent standard deviation from three biological replicates.

Supplementary Figure 2

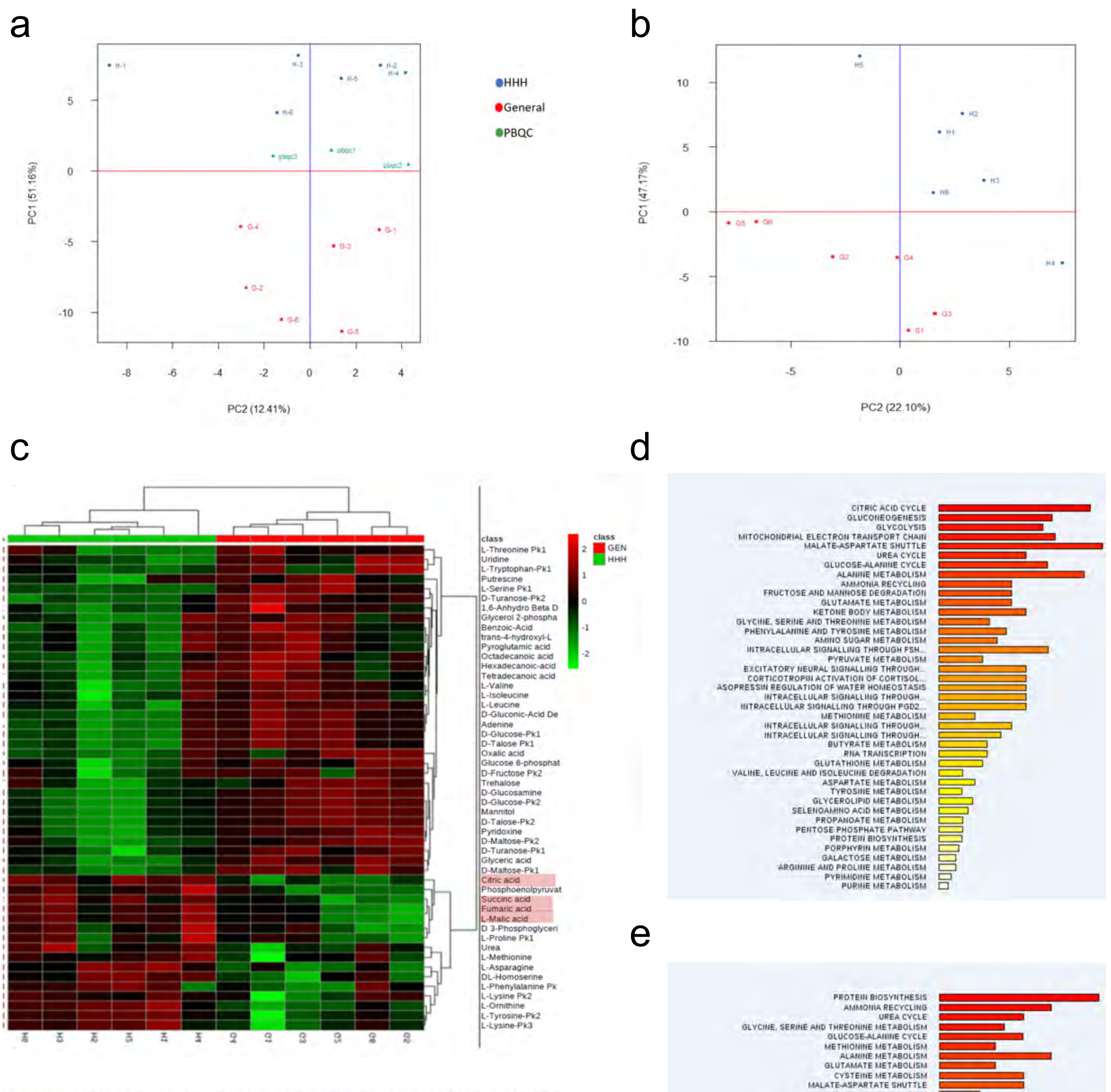

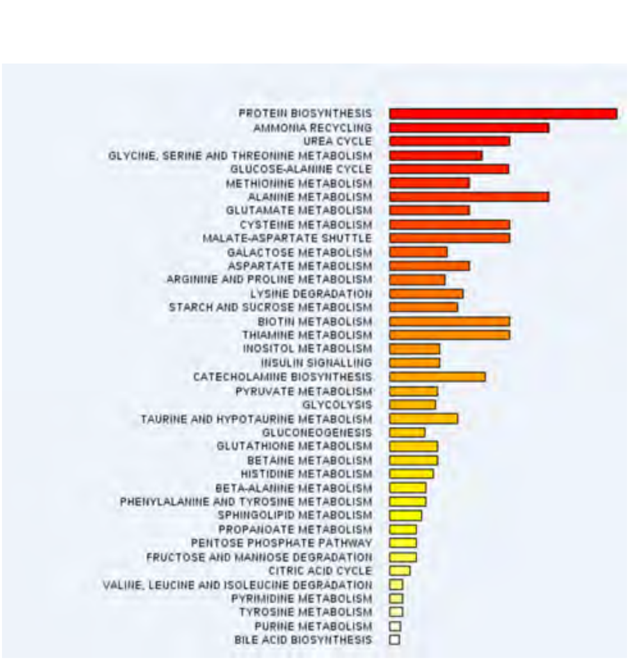

Supplementary Figure 2. Analysis of metabolomics data for the GCTM-2^{high}CD9^{high}EPCAM^{high} (HHH) and remaining (GEN) cells. a-b, unsupervised unbiased principal component analysis of metabolite levels from LC-MS (a) and GC-MS (b) data. PBQC are peripheral blood mononuclear cells used as a control. c, heatmap display of enrichment of metabolites in HHH cells versus GEN population determined by GC-MS analysis. d-e, metabolic pathway analysis of differential metabolite content in the HHH (d) or GEN (e) population by LC-MS.

Supplementary Figure 3

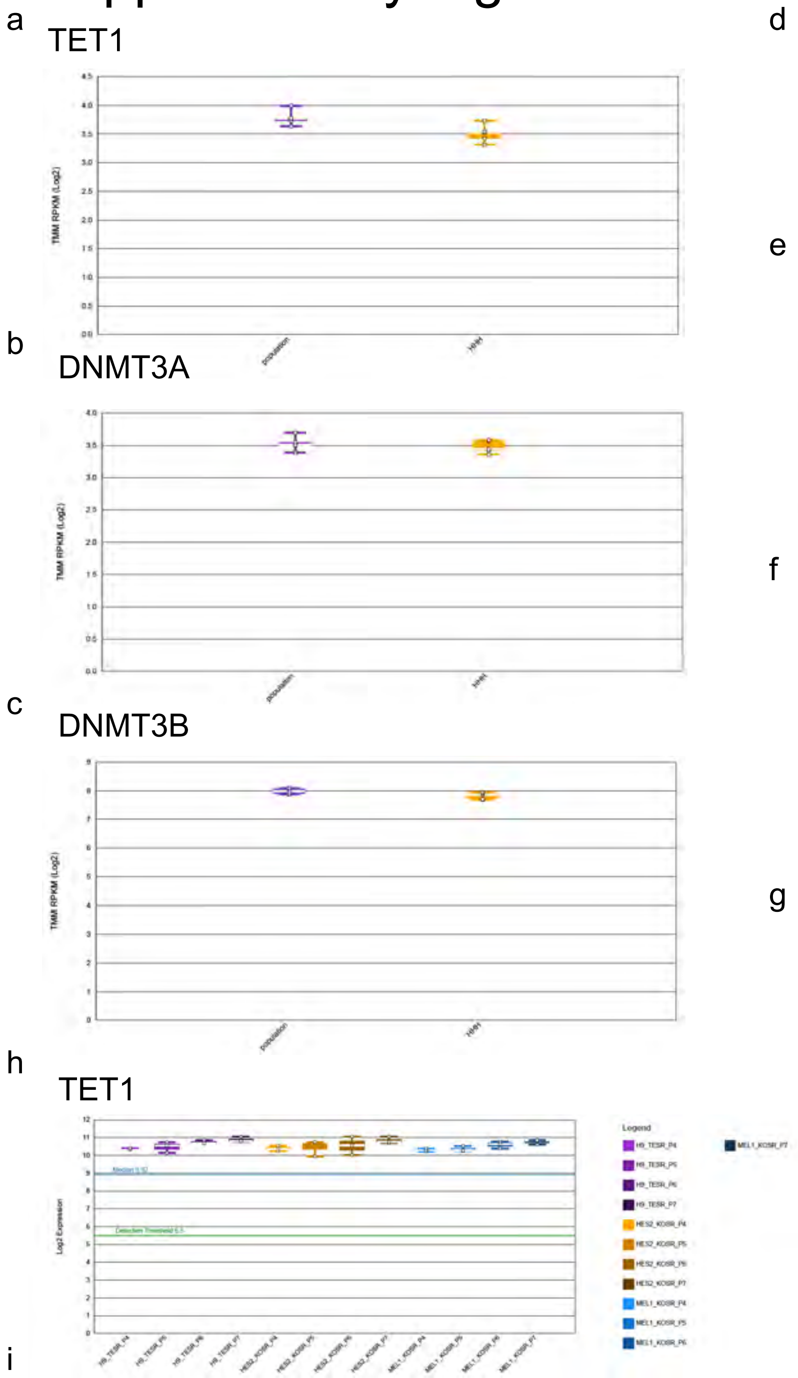

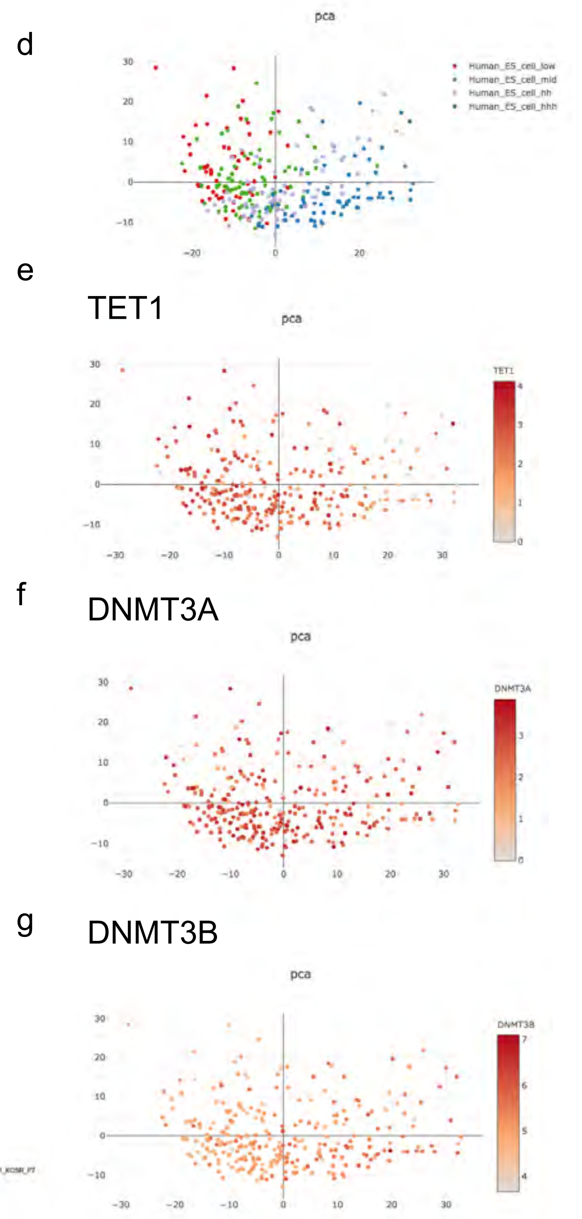

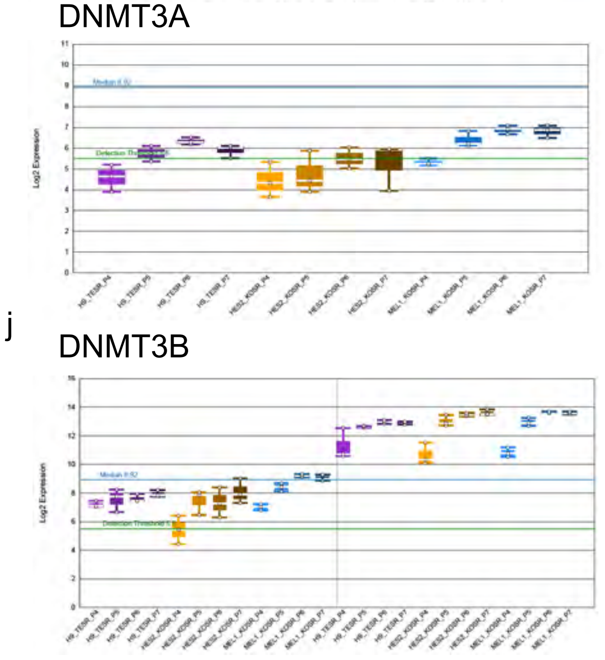

Supplementary Figure 3. Expression of *TET1*, *DNMT3A*, and *DNMT3B* in subpopulations of hPSC. Figure shows expression of *TET1*, *DNMT3A*, and *DNMT3B* in hPSC subpopulations by RNA-seq on bulk subpopulations (a-c) or single cells (d-g) in this study, and in the previous study of Kolle et al. (27) using a similar flow cytometry fractionation strategy (h-j). a-c, expression levels in unsorted (purple symbols) or GCTM-2^{high}CD9^{high}EPCAM^{high} (orange) symbols. Values in log_2 TMM RPKM. d, principal component analysis of single hPSC cell gene expression by subpopulation (low, red symbols, GCTM-2^{low}CD9^{low}; mid, green symbols, GCTM-2^{mid}CD9^{mid}; hh, grey symbols, GCMT-2^{high}CD9^{high}; hhh, blue symbols, GCTM-2^{high}CD9^{high}EPCAM^{high}). e-g, single cell expression of the three genes relative to position of cell in PCA in d. h-i, microarray gene expression data on three hPSC cell lines (WA09 grown in defined mTeSR medium, ESI-02 and MEL1 grown in Knockout Serum Replacer in the presence of mouse embryo fibroblast feeder cells), and sorted into four populations showing highest (P7) to lowest (P4) expression of cell surface antigens GCTM-2 and CD9 (27). Values are mean log_2 expression +/- standard deviation of three biological replicates.

Supplementary Figure 4

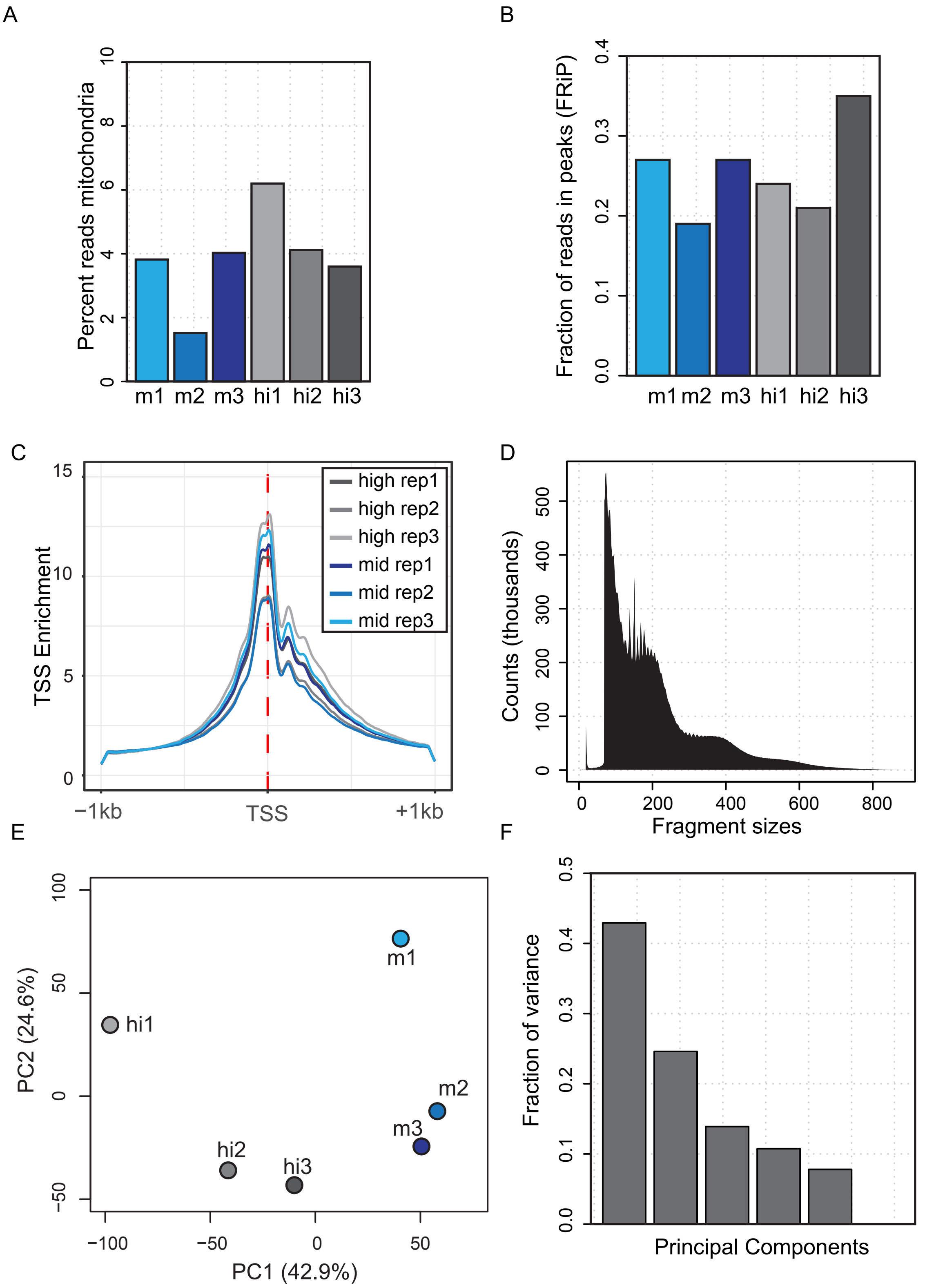

Supplementary Figure 4. ATAC-seq library quality control. A, Percent of reads mapped to mitochondria. FAST-ATAC protocol was developed to reduce the proportion of mitochondria to the total library. b, fraction of reads mapped to peak regions for each replicate. C, aggregate ATAC-seq signal surrounding all unique annotated TSS sites. d, distribution of fragment insert sizes from GCTM-2^{mid}CD9^{mid} replicate 1. High quality libraries have a large number of sequence lengths from nucleosome depleted regions (< 150 bp), and clear mono- and di-nucleotide sequences. e, scatterplot of the first two principle components of all open chromatin regions. f, barplot of variance explained for each principle component. m and mid and hi and high refer to GCTM-2^{mid}CD9^{mid} and GCTM-2^{high}CD9^{high}EPCAM^{high} replicates respectively.

Supplementary Figure 5

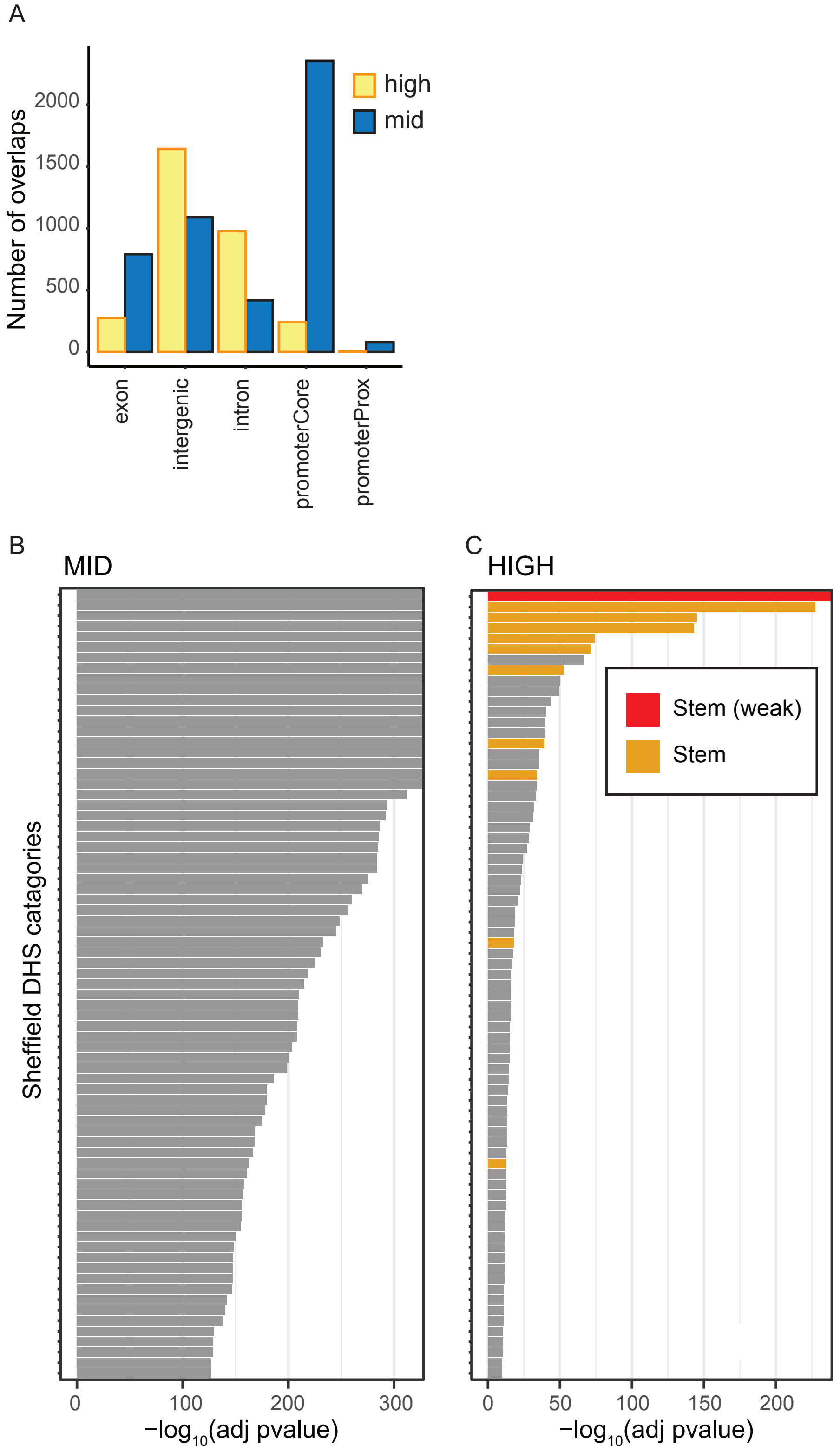

Supplementary Figure 5. Regions of increased DNA accessibility in GCTM-2^{high}CD9^{high}EPCAM^{high} (high) population are enriched for stem cell-specific chromatin sites. a, bar chart of differential open chromatin regions between GCTM-2^{high}CD9^{high}EPCAM^{high} (high, yellow) and GCTM-2^{mid}CD9^{mid} (mid, blue) populations showing overlap with annotated genomic features. GCTM-2^{high}CD9^{high}EPCAM^{high} peaks are found in intergenic and intronic regions, while GCTM-2^{mid}CD9^{mid} peaks are more frequently found at promoters. b and c, bar chart of 75 most significant enriched overlaps of GCTM-2^{mid}CD9^{mid} (b) and GCTM-2^{high}CD9^{high}EPCAM^{high} (c) population with the Sheffield DNAse hypersensitivity clusters. Overlap with stem cell specific clusters is indicated in orange. Red bar is annotated as "stem cell specific (weak)".

Supplementary Figure 6

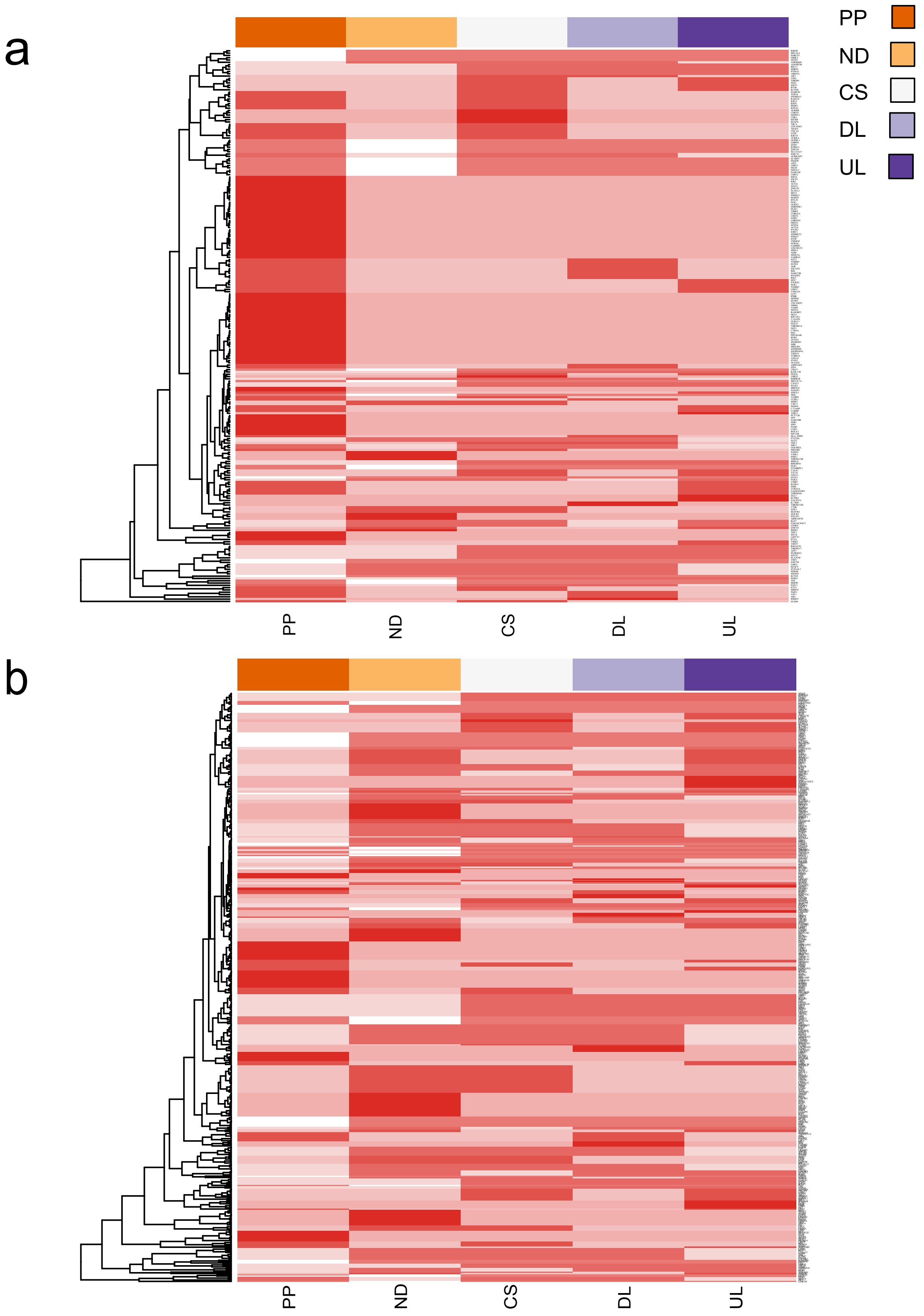

Supplementary Figure 6. Heatmaps showing distribution of differentially expressed genes in GCTM-2^{high}CD9^{high}EPCAM^{high} (a) and unsorted cells (general population, GEN, b) across the cortical neural differentiation time course described in Cortecon (29). PP, pluripotency; ND, neural differentiation; CS, cortical specification; DL, deep layer; UL, upper layer.

Supplementary Figure 7

LHX5

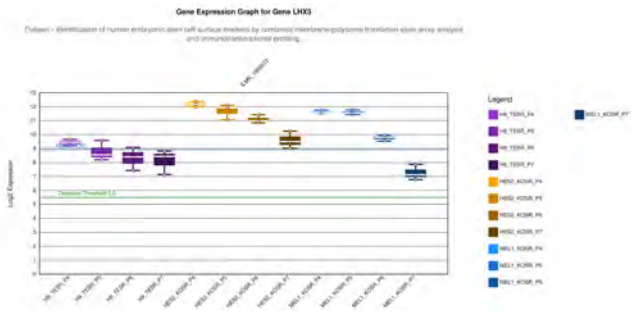

TNFRSF19

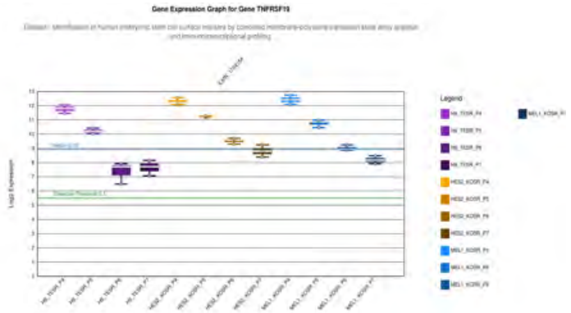

FST

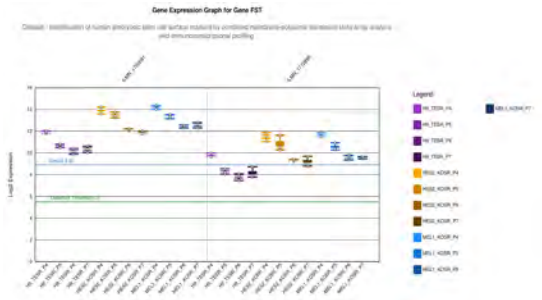

CDH6

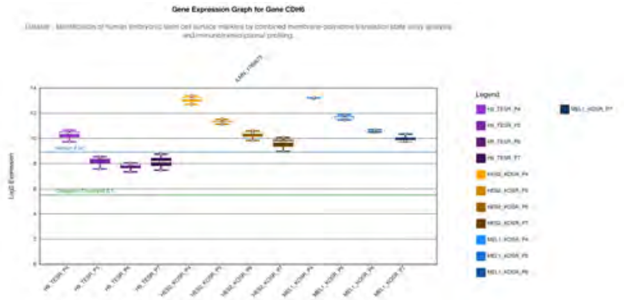

FRZB

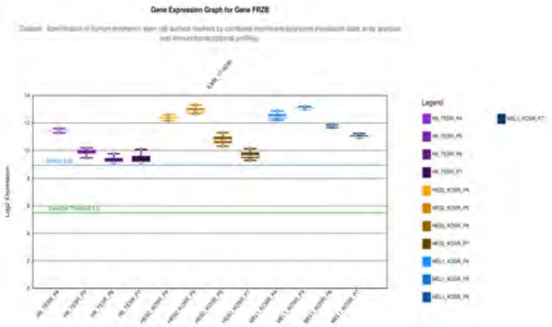

LRP2

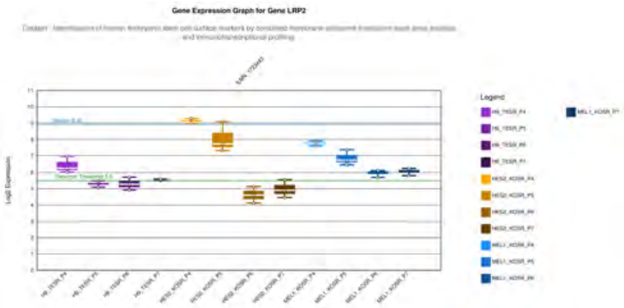

GREB1L

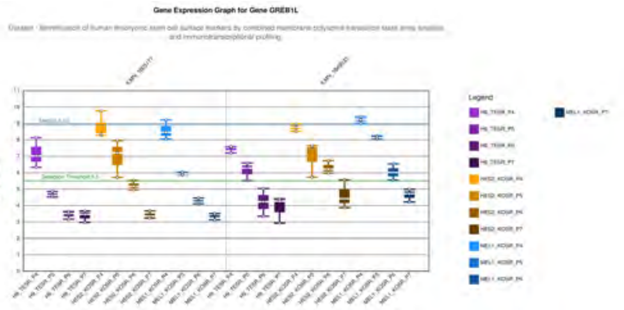

LIXL1

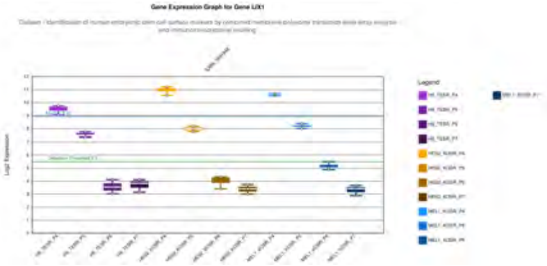

SDK2

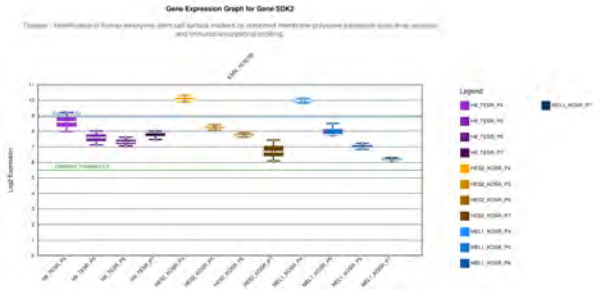

Supplementary Figure 7. Data from RNA microarray analyses in [1] showing expression in stem cell subpopulations of genes upregulated in the General population in this work. Data from three stem cell lines grown under defined conditions or in medium containing serum replacement with feeder cells are shown.

1. Kolle G, Ho M, Zhou Q, Chy HS, Krishnan K, Cloonan N, Bertoncello I, Laslett AL, Grimmond SM: **Identification of human embryonic stem cell surface markers by combined membrane-polysome translation state array analysis and immunotranscriptional profiling.** *Stem Cells* 2009, **27:**2446-2456.

Supplementary Figure 8

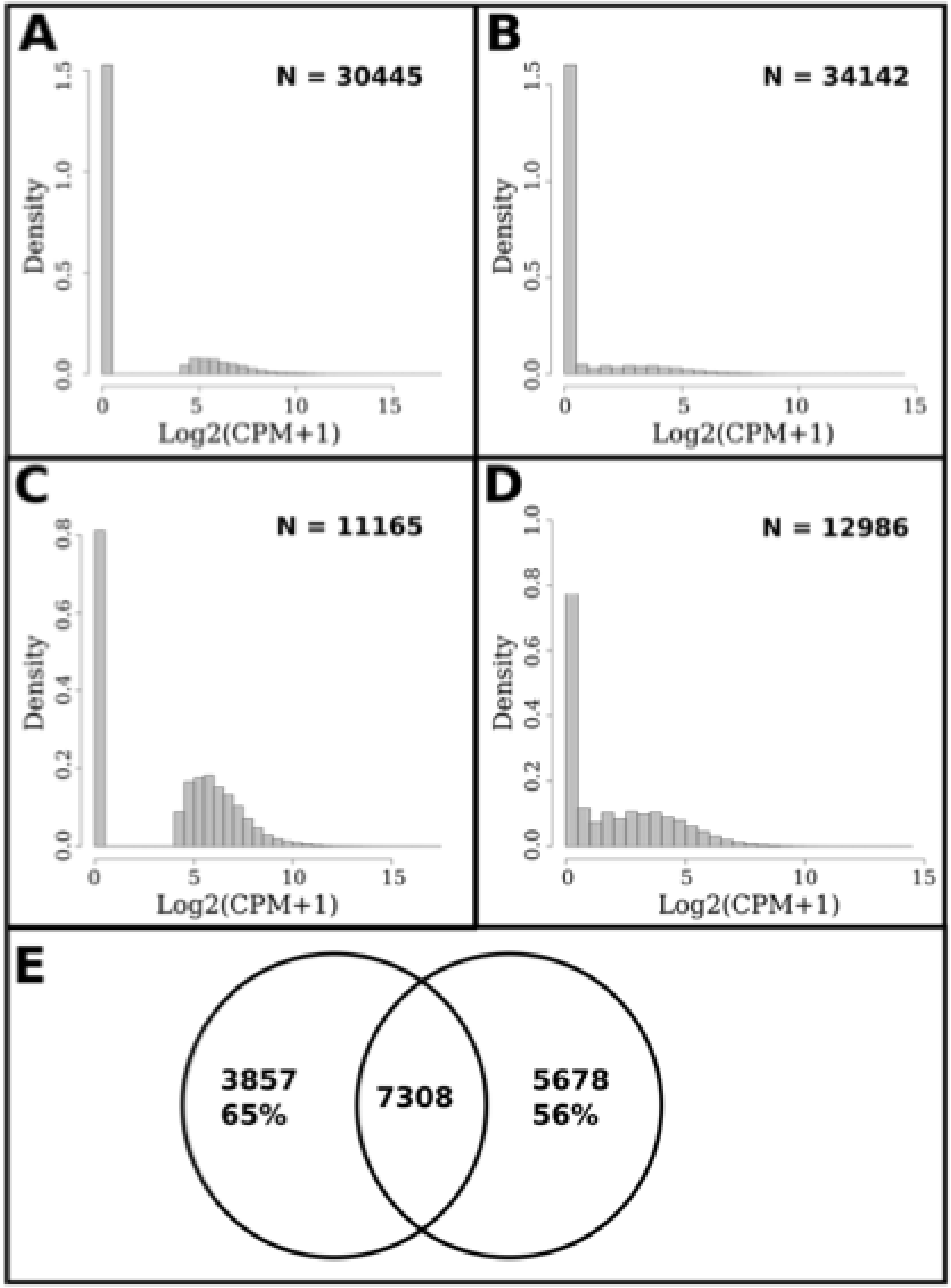

Supplementary Figure 8. Distribution of gene expression in processed single cell RNA-seq for human and non-human primate datasets. a, histogram displaying expression of all 30445 genes in the human scRNA-seq data. X-axis represents Log2(CPM+1) expression and Y-axis represents density. b, histogram displaying expression of all 34142 genes in the non-human primate scRNA-seq data. X-axis represents Log2(CPM+1) expression and Y-axis represents density. C, histogram of the 11165 genes expressed above 5 Log2(CPM+1) in at least 10% of cells classified in a given phenotype in the human scRNA-seq data. Xaxis represents Log2(CPM+1) expression and Y-axis represents density. d, histogram of the 12986 genes expressed above 1 Log2(CPM+1) in at least 10% of cells classified in a given phenotype in the non-human primate scRNA-seq data. X-axis represents Log2(CPM+1) expression and Y-axis represents density. e, Venn diagram displaying the overlap of expressed genes between human (left) and non-human primate (right) gene-filtered datasets.

Supplementary Figure 9

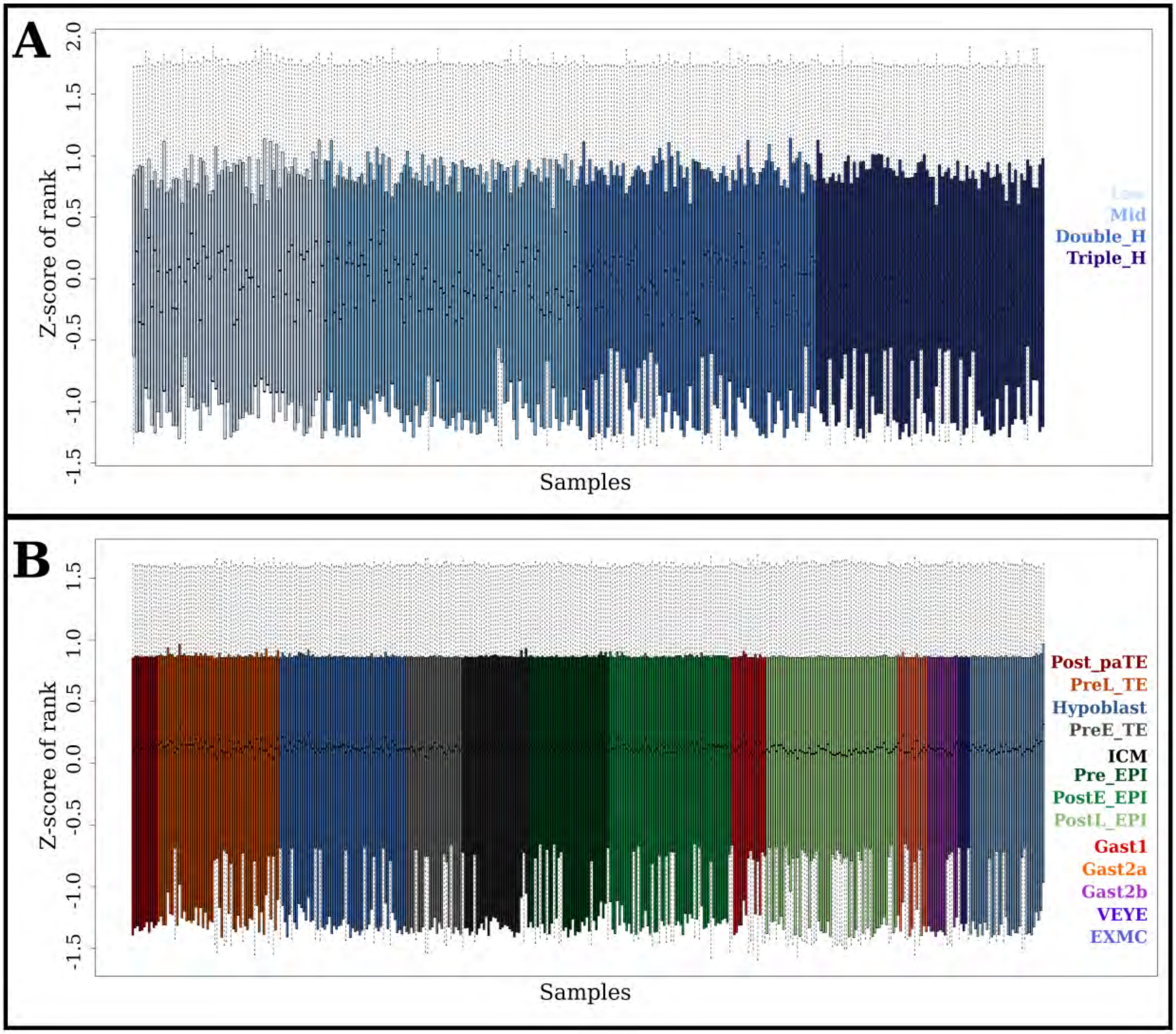

Supplementary Figure 9. Z-scores of ranked expression per cell. a, box plots indicating z scores of ranked gene expression in each cell of the human dataset. X-axis represents sample and Y-axis represents Z-score of ranked expression. Color indicates cell phenotype. The midline point of each colored box represents the median, with upper and lower limits of the colored box describing the 75th and 25th percentile respectively. Whiskers extend up to 1.5 times the interquartile range. b, box plots indicating z scores of ranked gene expression in each cell of the non-human primate dataset. X-axis represents sample and Y-axis represents Z-score of ranked expression. Color indicates cell phenotype. The midline point of each colored box represents the median, with upper and lower limits of the colored box describing the 75th and 25th percentile respectively. Whiskers extend up to 1.5 times the interquartile range.

Supplementary Figure 10

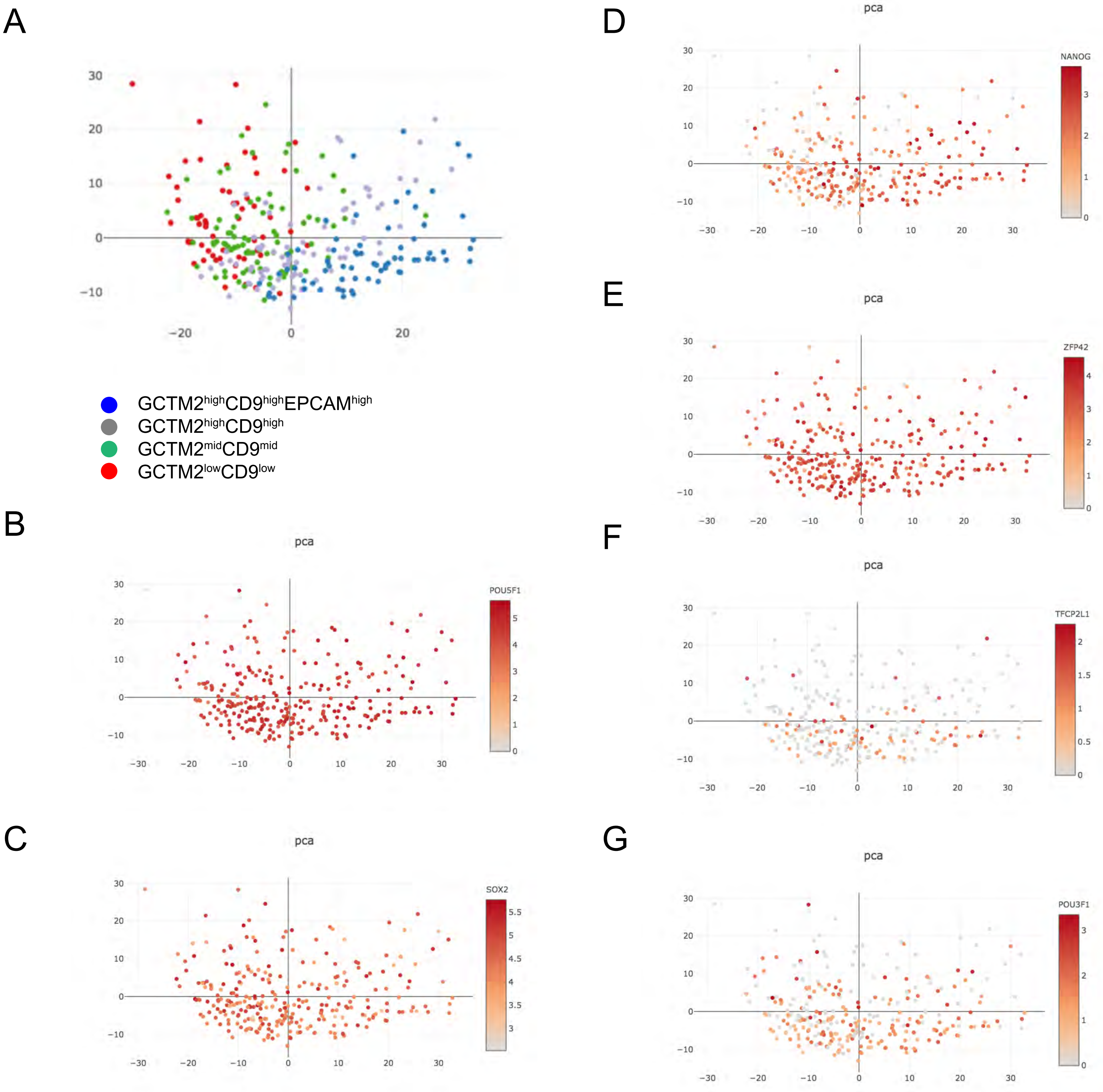

Supplementary Figure 10. Single cell gene expression analysis for pluripotency associated transcription factors in subsets of hPSC. a, principal component analysis of cell subsets. b, POU5F1. c, SOX2. d, NANOG. e, ZFP42. f, TFCP2L1. G, POU3F1.

Supplementary Figure 11

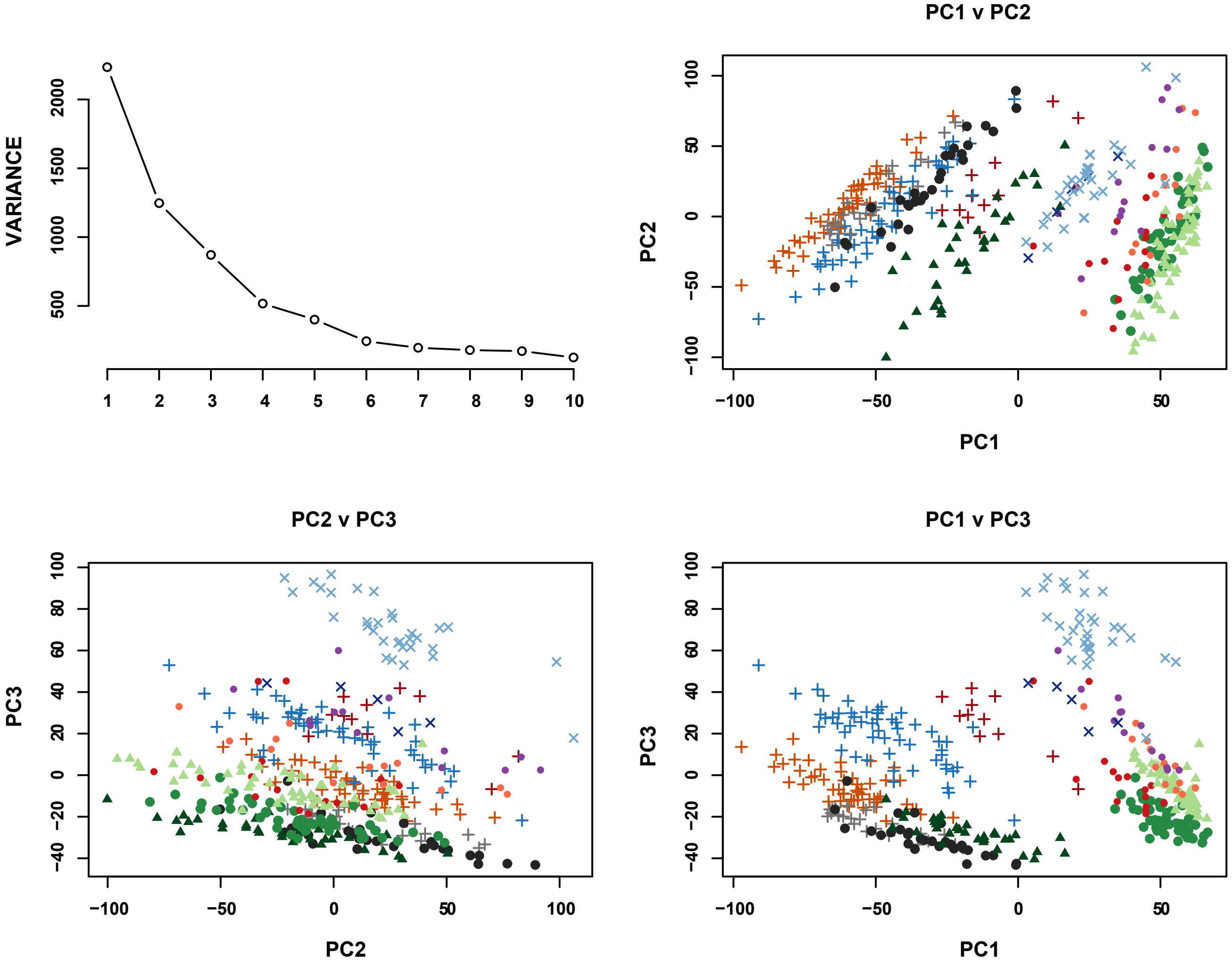

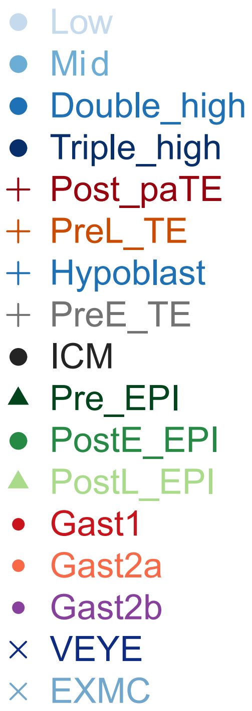

Supplementary Figure 11. Principal component analysis of single cell RNA-seq cynomolgus embryo data from [30]; single embryo cells classified according to [30]. Top left, Screeplot demonstrating the amount of variability in the data accounted for by each component; top right, graph displaying data distribution along first and second components; bottom left; graph displaying data distribution along the second and third components; bottom right, graph displaying data distribution along the second and third components. Color and shape of point indicate sample phenotype, each point representing a single cell.

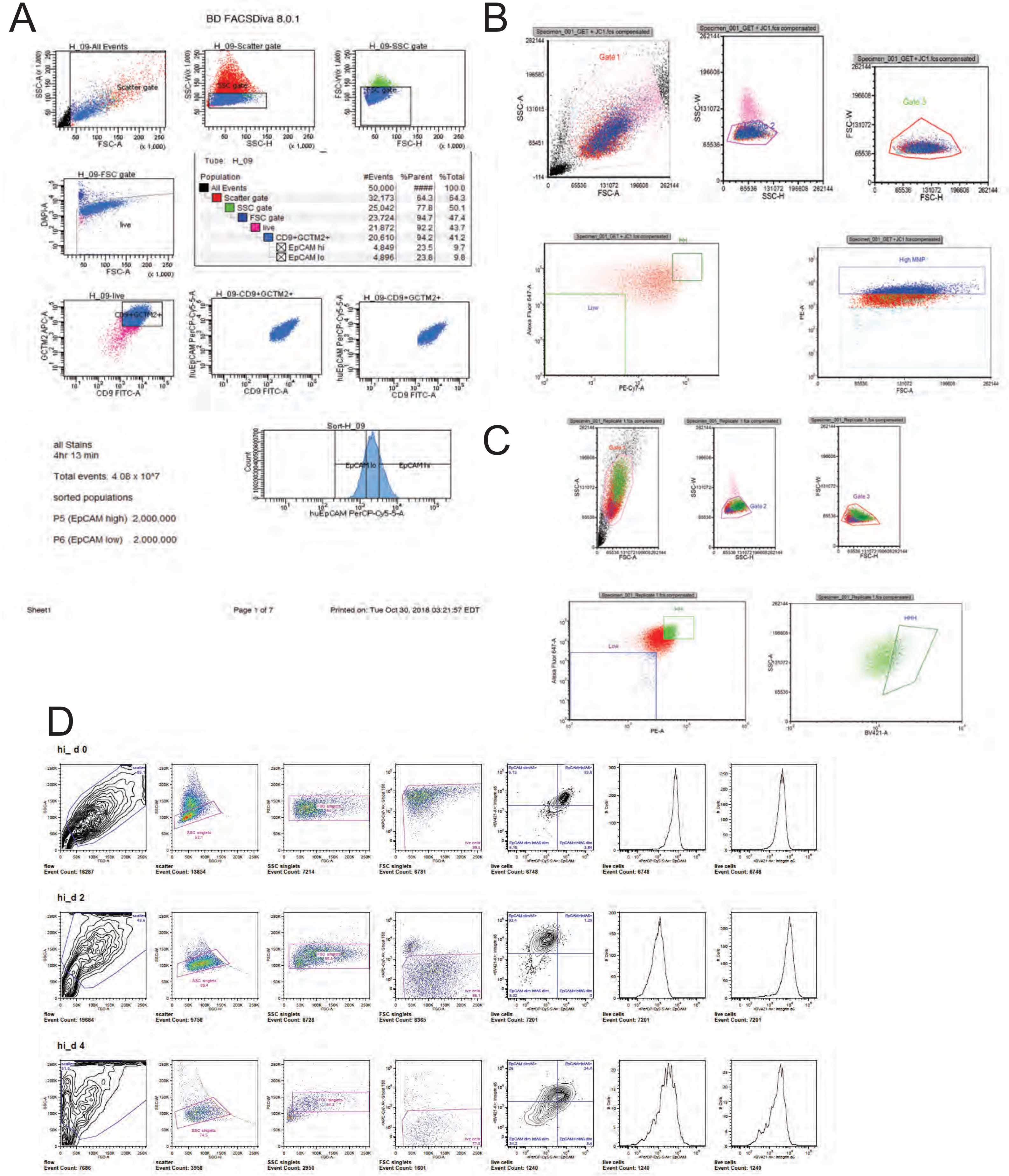

Supplementary Figure 12. Exemplary flow cytometry gating strategies for this study. a, strategy for isolation of GCTM-2$_{high}$CD9$_{high}$EPCAM$_{high}$ cells (Figures 2-9). b, strategy for isolation of GCTM-2$_{high}$CD9$_{high}$ and GCTM-2$_{low}$CD9$_{low}$ cells and determination of mitochondrial membrane potential with JC1 or TMRM (Figure 3). c, strategy for enumeration of ITGA6$_{+}$/EPCAM$_{+}$ cells (Figure 2).

Supplementary Table 1. LC-MS analysis showing metabolites that differed significantly between GCTM-2$_{high}$CD9$_{high}$EPCAM$_{high}$ subpopulation and remaining sorted cells (general population). Table lists metabolites that are significantly changed in cell extracts of GCTM-2$_{high}$CD9$_{high}$EPCAM$_{high}$ and general populations (in order of significance). Blue rows show metabolites that were higher in HHH population while orange rows show metabolites that were higher in general population. A two-sided Student's t-test was performed with alpha = 0.05. This was followed by correcting for multiple comparisons using the Benjamini–Hochberg method for controlling false discovery rate with a false discovery threshold of 0.05.

Metabolite	p-value	BH Adjusted p-value	Log2 fold change	ID type
Isocitric acid	1.32E-07	1.22E-05	0.524642314	Absolute
Glutamine	1.93E-06	8.87E-05	-0.607704314	Absolute
Citric acid	6.12E-06	0.000164247	0.586430691	Absolute
4-Hydroxyproline	1.49E-05	0.000164247	0.527091672	Absolute
trans-4-Hydroxy-L-proline	1.59E-05	0.000164247	0.526079094	Absolute
Cystathionine	1.00E-05	0.000164247	0.351659058	Absolute
L-Asparagine	1.61E-05	0.000164247	-0.388721353	Absolute
Glycylglycine	1.61E-05	0.000164247	-0.388721353	Absolute
Oxalic acid	1.51E-05	0.000164247	-0.47123236	Absolute
5-Aminolevulinate	1.85E-05	0.000170312	0.523920154	Absolute
L-2-Aminoadipate	2.30E-05	0.000192261	-0.447839981	Absolute
D-Ribose	3.25E-05	0.00019939	-0.727401163	Absolute
D-Xylose	3.25E-05	0.00019939	-0.727401163	Absolute
D-Fructose	3.18E-05	0.00019939	-0.767959474	Absolute
(R)-Lactate	3.19E-05	0.00019939	-0.774077624	Absolute
Aspartic acid	4.24E-05	0.000243685	-0.385552536	Absolute
3-Phosho-D-glycerate	4.53E-05	0.000245002	0.482504025	Absolute
2-oxobutanoate	5.28E-05	0.00026967	-0.785205959	Absolute

myo-Inositol	6.30E-05	0.000304823	-0.818377372	Absolute
Succinate semialdehyde	7.01E-05	0.000322611	-0.78868289	Absolute
(R)-2-Hydroxyglutarate	9.84E-05	0.000413145	0.333953643	Absolute
N2-Acetyl-L-lysine	9.88E-05	0.000413145	-1.132906293	Absolute
Succinic acid	0.0001043	0.000417093	0.506152229	Absolute
D-Glucose	0.0001163	0.000445913	-0.847995972	Absolute
2-Ketoglutaric acid	0.0003023	0.001069512	0.585100303	Absolute
Methylmalonate	0.0002976	0.001069512	0.510621202	Absolute
Acetoacetate	0.0003189	0.0010865	0.521594149	Absolute
D-Glucuronate	0.0005034	0.001596855	-0.330989736	Absolute
D-Galacturonate	0.0005034	0.001596855	-0.330989736	Absolute
Thiamin	0.0008007	0.00245549	-0.770038699	Absolute
Arginine	0.001096	0.003252568	-0.558114321	Absolute
Ribitol	0.0014502	0.004042845	0.330140832	Absolute
X-D-Galactono-1-4-lactone-	0.0019808	0.005359799	-0.409347552	Absolute
Fumaric acid	0.0031647	0.008122226	0.425029275	Absolute
Cystine	0.0031783	0.008122226	-0.826901918	Absolute
Deoxyinosine monophosphate	0.0035506	0.008828582	-0.310533314	Absolute
Orotidine	0.003765	0.009115318	0.255350639	Absolute
D-Fructose-6-phosphate	0.0040313	0.009509659	0.257580698	Absolute
Malic acid	0.0048468	0.011048939	0.47075813	Absolute
L-Alanine	0.0049772	0.011048939	0.236019278	Absolute
Uridine diphosphate-N-acetyl-D-glucosamine	0.0050441	0.011048939	0.212823099	Absolute
Adenosine-5'-triphosphate	0.0063666	0.013621667	0.26062801	Absolute
L-Threonine	0.0077217	0.016145394	-0.252537882	Absolute
Homoserine	0.0079236	0.016199384	-0.251633673	Absolute

Nicotinamide adenine dinucleotide- reduced (NADH)	0.0096662	0.019332382	0.356589657	Absolute
Serine	0.0114596	0.02243154	-0.306785283	Absolute
Glutamic acid	0.012018	0.023034515	-0.169676121	Absolute
Uridine diphosphate	0.0126088	0.023349968	0.571412567	Absolute
Phosphoenolpyruvic acid	0.0126902	0.023349968	0.223702917	Absolute
L-Valine	0.0154158	0.02780894	-0.368588803	Absolute
L-Tyrosine	0.0164475	0.029099389	-0.290497601	Absolute
Glutathione	0.016802	0.029165807	0.438737885	Absolute
Betaine	0.0176997	0.030154962	-0.355622103	Absolute
Pyruvate	0.0197157	0.032979039	-0.731569295	Absolute
Lysine	0.021583	0.035457726	-0.372573214	Absolute
Mannitol	0.0248276	0.040072586	-0.311368405	Absolute
Taurine	0.0288944	0.045832447	-0.225013689	Absolute
Guanosine diphosphate-mannose	0.0319557	0.049829214	0.293422161	Absolute
**D-Sorbitol**	**0.0467135**	**0.070453141**	**0.673218477**	**Absolute**

Supplementary Table 2. GC-MS analysis showing metabolites that differed significantly between GCTM-2_{high}CD9_{high}EPCAM$_{high}$ subpopulation and remaining sorted cells (general population). Table lists metabolites that are significantly changed in cell extracts of GCTM-2_{high}CD9_{high}EPCAM$_{high}$ and general populations (in order of significance). Blue rows show metabolites that were higher in HHH population while orange rows show metabolites that were higher in general population. A two-sided Student's t-test was performed with alpha = 0.05. This was followed by correcting for multiple comparisons using the Benjamini–Hochberg method for controlling false discovery rate with a false discovery threshold of 0.05.

Metabolite	p-value	BH Adjusted p-value	Log2 fold change	ID type
L-Ornithine	0.000517	0.005339	1.849315	Absolute
Citric acid	0.000248	0.005339	0.859864	Absolute
Glyceric acid	0.000439	0.005339	-0.47783	Absolute
D-Maltose-Peak1	0.000607	0.005339	-0.4996	Absolute
D-Turanose-Peak2	0.000314	0.005339	-0.73448	Absolute
D-Glucosamine	0.000294	0.005339	-0.8207	Absolute
D-Glucose-Peak2	0.000636	0.005339	-0.82803	Absolute
Mannitol	0.000594	0.005339	-0.86019	Absolute
D-Talose-Peak2	0.000592	0.005339	-0.86062	Absolute
L-Serine	0.000459	0.005339	-1.77365	Absolute
D-Maltose-Peak2	0.00183	0.013972	-0.45887	Absolute
Pyridoxal	0.002407	0.016078	-0.62319	Absolute
D-Gluconic-Acid-Delta-Lactone	0.002488	0.016078	-0.68587	Absolute
Succinic acid	0.002957	0.017741	0.478017	Absolute
D-Glucose-Peak1	0.003551	0.019883	-0.62562	Absolute
Phosphoenolpyruvic acid	0.005606	0.023978	0.380612	Absolute
D-Talose-Peak1	0.005468	0.023978	-0.60396	Absolute
D-Turanose-Peak1	0.005709	0.023978	-0.62604	Absolute
Adenine	0.004782	0.023978	-0.639	Absolute
Uridine	0.005198	0.023978	-1.08842	Absolute
L-Asparagine	0.0067	0.025582	1.113448	Absolute
Fumaric acid	0.006432	0.025582	0.467122	Absolute
Malic acid	0.008822	0.03222	0.476851	Absolute
Octadecanoic acid	0.00961	0.033634	-0.50692	Absolute
L-Tyrosine	0.011051	0.035584	1.622878	Absolute

Lysine	0.012264	0.035584	1.560901	Absolute
DL-Homoserine	0.012285	0.035584	1.435386	Absolute
L-Leucine	0.01061	0.035584	-0.30289	Absolute
1-6-Anhydro-Beta-D-Glucose (Levoglucosan)	0.011904	0.035584	-0.59403	Absolute
Oxalic acid	0.015423	0.043091	-0.38435	Absolute
Hexadecanoic acid	0.015903	0.043091	-0.49781	Absolute
**Benzoic acid**	**0.022223**	**0.058336**	**-0.46482**	**Absolute**
**Trehalose**	**0.025241**	**0.062361**	**-0.28773**	**Absolute**
**L-Threonine**	**0.024974**	**0.062361**	**-0.83173**	**Absolute**
**D-Glucose 6-phosphate**	**0.026682**	**0.064036**	**-0.4393**	**Absolute**
**L-Valine**	**0.028355**	**0.066162**	**-0.26637**	**Absolute**
**L-Tryptophan**	**0.032925**	**0.074749**	**-0.52234**	**Absolute**
**Glycerol 2-phosphate**	**0.03931**	**0.086896**	**-0.83679**	**Absolute**

Supplementary Table 3. Pathway analysis of differentially expressed genes in GCTM-2_{high}CD9_{high}EPCAM$_{high}$ subpopulation and unsorted cells (general population) identified by RNA-seq.

GO Analysis with Panther.db (Slim)

Biological Process	Homo sapiens - REFLIST (21042	Client Text Box Input (548)	Client Text Box Input (expected)	Client Text Box Input (fold Enrichment)	Client Text Box Input (FDR)
protein folding (GO:0006457)	94	14	2.45	5.72	1.87E-05
translation (GO:0006412)	198	27	5.16	5.24	5.90E-09
rRNA metabolic process (GO:0016072)	113	15	2.94	5.1	2.03E-05
oxidative phosphorylation (GO:0006119)	47	6	1.22	4.9	1.47E-02
mitochondrion organization (GO:0007005)	92	11	2.4	4.59	8.37E-04
chromosome segregation (GO:0007059)	96	9	2.5	3.6	1.20E-02
generation of precursor metabolites and energy (GO:000609	171	15	4.45	3.37	1.09E-03
respiratory electron transport chain (GO:0022904)	107	9	2.79	3.23	1.81E-02
protein targeting (GO:0006605)	171	14	4.45	3.14	2.99E-03
nuclear transport (GO:0051169)	123	9	3.2	2.81	3.50E-02
cellular component biogenesis (GO:0044085)	776	55	20.21	2.72	7.55E-09
RNA splicing, via transesterification reactions (GO:0000375)	156	11	4.06	2.71	2.08E-02
mRNA splicing, via spliceosome (GO:0000398)	178	12	4.64	2.59	2.07E-02
mRNA processing (GO:0006397)	244	16	6.35	2.52	9.53E-03
regulation of cell cycle (GO:0051726)	186	12	4.84	2.48	2.68E-02
mitosis (GO:0007067)	231	14	6.02	2.33	3.20E-02
protein complex assembly (GO:0006461)	244	14	6.35	2.2	4.01E-02
protein complex biogenesis (GO:0070271)	245	14	6.38	2.19	4.02E-02
cell cycle (GO:0007049)	723	39	18.83	2.07	7.45E-04
organelle organization (GO:0006996)	1212	62	31.56	1.96	2.01E-05
cellular component organization or biogenesis (GO:0071840)	2099	104	54.66	1.9	2.70E-08
biosynthetic process (GO:0009058)	1745	86	45.45	1.89	8.06E-07
protein localization (GO:0008104)	532	26	13.85	1.88	2.12E-02
intracellular protein transport (GO:0006886)	694	33	18.07	1.83	1.24E-02
protein transport (GO:0015031)	736	34	19.17	1.77	1.50E-02
RNA metabolic process (GO:0016070)	1570	72	40.89	1.76	9.99E-05
cellular component organization (GO:0016043)	1964	87	51.15	1.7	3.83E-05
nucleobase-containing compound metabolic process (GO:0C	2797	122	72.84	1.67	7.77E-07
protein metabolic process (GO:0019538)	1583	69	41.23	1.67	6.73E-04
nitrogen compound metabolic process (GO:0006807)	2524	104	65.73	1.58	6.06E-05
catabolic process (GO:0009056)	1176	48	30.63	1.57	1.75E-02
phosphate-containing compound metabolic process (GO:00C	1595	63	41.54	1.52	1.17E-02
primary metabolic process (GO:0044238)	4753	187	123.78	1.51	6.39E-08
metabolic process (GO:0008152)	5878	227	153.08	1.48	2.97E-09
cellular process (GO:0009987)	8247	273	214.78	1.27	1.81E-05
Unclassified (UNCLASSIFIED)	10206	220	265.8	0.83	1.26E-03
biological regulation (GO:0065007)	2985	54	77.74	0.69	2.06E-02
cell communication (GO:0007154)	2686	46	69.95	0.66	1.25E-02
response to stimulus (GO:0050896)	2677	44	69.72	0.63	6.63E-03
single-multicellular organism process (GO:0044707)	1665	24	43.36	0.55	1.21E-02
multicellular organismal process (GO:0032501)	1684	24	43.86	0.55	1.10E-02
cell surface receptor signaling pathway (GO:0007166)	1206	12	31.41	0.38	1.23E-03
system process (GO:0003008)	1020	10	26.56	0.38	3.52E-03
neurological system process (GO:0050877)	924	9	24.06	0.37	8.05E-03
immune system process (GO:0002376)	669	3	17.42	0.17	7.25E-04
immune response (GO:0006955)	383	0	9.97	< 0.01	1.20E-03
sensory perception of smell (GO:0007608)	240	0	6.25	< 0.01	2.06E-02
sensory perception of chemical stimulus (GO:0007606)	282	0	7.34	< 0.01	1.19E-02
**Cellular Component**	**Homo sapiens - REFLIST (21042**	**Client Text Box Input (548)**	**Client Text Box Input (expected)**	**Client Text Box Input (fold Enrichment)**	**Client Text Box Input (FDR)**
ribosome (GO:0005840)	160	48	4.17	11.52	7.25E-31
proton-transporting ATP synthase complex (GO:0045259)	19	4	0.49	8.08	8.81E-03
ribonucleoprotein complex (GO:0030529)	427	77	11.12	6.92	5.69E-36
cytosol (GO:0005829)	511	66	13.31	4.96	6.58E-24
mitochondrial inner membrane (GO:0005743)	108	10	2.81	3.56	3.67E-03
nucleolus (GO:0005730)	117	9	3.05	2.95	1.65E-02
mitochondrion (GO:0005739)	392	29	10.21	2.84	7.74E-06
macromolecular complex (GO:0032991)	2156	158	56.15	2.81	4.62E-31
microtubule (GO:0005874)	137	9	3.57	2.52	3.77E-02
nuclear outer membrane-endoplasmic reticulum membrane r	234	15	6.09	2.46	6.93E-03
endoplasmic reticulum (GO:0005783)	412	24	10.73	2.24	2.32E-03
cytoplasm (GO:0005737)	3171	180	82.58	2.18	1.68E-23
organelle (GO:0043226)	3910	197	101.83	1.93	2.99E-20
intracellular (GO:0005622)	5262	261	137.04	1.9	4.85E-28

cell part (GO:0044464)	5531	265	144.04	1.84	2.01E-26
protein complex (GO:0043234)	1796	86	46.77	1.84	5.42E-07
nucleus (GO:0005634)	1943	81	50.6	1.6	2.11E-04
Unclassified (UNCLASSIFIED)	13830	267	360.18	0.74	6.73E-15
plasma membrane (GO:0005886)	1515	23	39.46	0.58	1.71E-02
extracellular region (GO:0005576)	673	4	17.53	0.23	1.25E-03
extracellular space (GO:0005615)	530	3	13.8	0.22	0.00451
**Molecular Function**	**Homo sapiens - REFLIST (21042**	**Client Text Box Input (548)**	**Client Text Box Input (expected)**	**Client Text Box Input (fold Enrichment)**	**Client Text Box Input (FDR)**
structural constituent of ribosome (GO:0003735)	126	38	3.28	11.58	1.75E-23
proton-transporting ATP synthase activity, rotational mechan	17	4	0.44	9.03	2.57E-02
hydrogen ion transmembrane transporter activity (GO:00150	72	9	1.88	4.8	4.50E-03
translation regulator activity (GO:0045182)	80	10	2.08	4.8	2.60E-03
kinase regulator activity (GO:0019207)	70	8	1.82	4.39	1.41E-02
structural molecule activity (GO:0005198)	506	51	13.18	3.87	2.20E-13
mRNA binding (GO:0003729)	142	13	3.7	3.52	4.05E-03
isomerase activity (GO:0016853)	103	9	2.68	3.36	2.85E-02
RNA binding (GO:0003723)	385	33	10.03	3.29	4.71E-07
nucleic acid binding (GO:0003676)	1625	64	42.32	1.51	1.95E-02
catalytic activity (GO:0003824)	4217	151	109.82	1.37	1.01E-03
binding (GO:0005488)	4911	164	127.9	1.28	9.72E-03
Unclassified (UNCLASSIFIED)	11852	236	308.66	0.76	5.55E-08

ICM	ICM<br>PRE EPI	ICM<br>PRE EPI	PRE EPI<br>POST EPI-E	PRE EPI<br>POST EPI	PRE EPI<br>POST EPI	POST EPI	POS EPI-L<br>GAST	GAST	GAST
AQP3	ANPEP	DNMT3L	DMRTB1	F2RL1	ANXA1	ARHGEF19	BMP4	EOMES	CER1
CLDN4	ANXA1	PRDM14	DNMT3L	FAM19A4	EVX1	BMP4	CHD7	FN1	EPHA4
ESRRB	CD53	SOX2	HIST1H1C	GABRB3	FEZ1	CAV1	HMGA2	GATA4	FGF17
IL6R	FGFR3		KHDC3L	GDF3	FGF4	CCND1	ITM2A	ID2	FOXA2
TBX3	GDF15		LGALS1	ITGA7	GABRB3	CCND2	SFRP1	MIXL1	GATA6
XIST	KHDC1L		NLRP2	MYLIP	GBX2	FGF2	TPM1	PITX2	GSC
	KLF4		SPP1	NPY1R	GDF3	FST	WWC3	T	HHEX
	KLF5		TUBB6	PLP1	KHDC3L	GPC4	ZIC2		KLF5
	OOEP		ZYG11A	RNASET2	KRT19	HAS3			LHX1
	S100P			SFRP2	LEFTY1	HESX1			OTX2
	TFAP2C			SGK1	NODAL	KDR			PDGFRA
	TFCP2L1			SLAIN1	SALL2	SFRP1			SOX17
				SOX2	TCF7L1	SFRP2			
				USP44	WNT5B	SOX11			
				VAT1L	ZIC2				
					ZSCAN10				

Supplementary Table 4. Stage-specific differentially expressed genes. Genes were selected on the basis of specificity to particular stages of development, or to particular stage transitions. ICM, inner cell mass; ICM/PRE EPI, inner cell mass/pre-implantation epiblast; PRE-EPI/POST EPI-E, pre-implantation epiblast/early post-implantation epiblast; PRE EPI/POST EPI; pre-implantation and post-implantation epiblast; POST EPI, post-implantation epiblast; POST EPI-L/GAST, late post-implantation epiblast and gastrulation; GAST, gastrulation.

			SINGLE CELLS				AGGREGATES		
		# OF CELLS	2000	1000	200	#OF AGGs	1000	500	100
**HH**									
	EXPT 1		165	16	0		344	50	0
	EXPT 2		161	28	0		309	82	0
	EXPT 3		76	16	0.25		137	55	6.5
**MID**									
	EXPT1		35	4	0		84	3	0
	EXPT2		72	1	0		63	4	0
	EXPT3		33	4.2	0.5		46	16	1

Supplementary Table 5. Biological replicate data for assay in Figure 1e showing colony formation of single cell and cell aggregates from GCTM-2_{high}CD9_{high} and GCTM-2_{mid}CD9_{mid} subpopulations. Table shows number of microcolonies formed after 4 days of culture by single cells (200, 1000, or 2000) or aggregates (100, 500, or 1000) of GCTM-2_{high}CD9_{high} (HS, HA) and GCTM-2_{mid}CD9_{mid} (MS, MA) subpopulations in 3 separate experiments. Values represent the mean from eight wells from each experiment.

Supplementary Table 6. Biological replicate data for cell cycle studies using Fucci reporter system shown in Supplementary Figure 1. Table shows proportion of cells in G1 for cultures grown in E8 defined medium without feeder cell support (four biological replicates), in serum replacement on mouse embryo fibroblasts (four biological replicates, or MTeSR defined medium without feeder cell support (six biological replicates).

FUCCI E8

	% in G1	Low	HH	HHH
	30012015	14.0	8.1	10.1
	02022015	8.6	3.3	3.3
	12022015	13.1	2.8	2.5
	31032015	13.4	9.9	11.8
Average		12.3	6.0	6.9
SD		2.5	3.5	4.7
SEM		1.2	1.8	2.4

FUCCI MEF

	% in G1	Low	HH	HHH
	30012015	57.2	24.6	18.0
	02022015	64.8	19.5	24.1
	16062015	69.0	4.3	3.4
	25062015	47.8	4.8	10.5
Average		59.7	13.3	14.0
SD		9.3	10.3	9.0
SEM		4.6	5.2	4.5

FUCCI MTeSR

	% in G1	Low	HH	HHH
	12005015	7.2	5.5	8.0
	19052015	13.6	9.0	12.3
	16062015	6.7	1.2	1.7
	25062015	9.2	1.0	1.0
	1072015	25.0	2.0	2.0
	9072015	6.3	0.4	0.6
Average		11.3	3.2	4.3
SD		7.2	3.4	4.8
SEM		2.9	1.4	2.0

	9072015	6.3	0.4	0.6
Average		11.3	3.2	4.3
SD		7.2	3.4	4.8
SEM		2.9	1.4	2.0
