# Supplementary material for: Unique properties of a subset of human pluripotent stem cells with high capacity for self-renewal
Source: Nat Commun. 2020 May 15;11:2420. doi: 10.1038/s41467-020-16214-8 (PMC7229198; doi:10.1038/s41467-020-16214-8)
Supplement: Supplementary file 3 — Reporting Summary [file 41467_2020_16214_MOESM3_ESM.pdf]

natureresearch

Corresponding author(s): MARTIN PERA

Last updated by author(s): Mar 30, 2020

Reporting Summary

Nature Research wishes to improve the reproducibility of the work that we publish. This form provides structure for consistency and transparency in reporting. For further information on Nature Research policies, see Authors & Referees and the Editorial Policy Checklist.

Statistics

For all statistical analyses, confirm that the following items are present in the figure legend, table legend, main text, or Methods section.

n/a	Confirmed	
☐	☒	The exact sample size (*n*) for each experimental group/condition, given as a discrete number and unit of measurement
☐	☒	A statement on whether measurements were taken from distinct samples or whether the same sample was measured repeatedly
☐	☒	The statistical test(s) used AND whether they are one- or two-sided<br>*Only common tests should be described solely by name; describe more complex techniques in the Methods section.*
☒	☐	A description of all covariates tested
☐	☒	A description of any assumptions or corrections, such as tests of normality and adjustment for multiple comparisons
☐	☒	A full description of the statistical parameters including central tendency (e.g. means) or other basic estimates (e.g. regression coefficient) AND variation (e.g. standard deviation) or associated estimates of uncertainty (e.g. confidence intervals)
☐	☒	For null hypothesis testing, the test statistic (e.g. *F*, *t*, *r*) with confidence intervals, effect sizes, degrees of freedom and *P* value noted<br>*Give P values as exact values whenever suitable.*
☒	☐	For Bayesian analysis, information on the choice of priors and Markov chain Monte Carlo settings
☒	☐	For hierarchical and complex designs, identification of the appropriate level for tests and full reporting of outcomes
☒	☐	Estimates of effect sizes (e.g. Cohen's *d*, Pearson's *r*), indicating how they were calculated

Our web collection on statistics for biologists contains articles on many of the points above.

Software and code

Policy information about availability of computer code

Data collection

DNA METHYLATION Sequence quality control was performed using FastQC [61]. Trimming of adapters and low-quality base calls was performed with trim_galore [62]. Trimmed reads were filtered for true RRBS reads (which contain an MspI cut site at the 5') using trimRRBSdiversityAdaptCustomers.py (NuGEN).
CHROMATIN ACCESSIBILITY Libraries were trimmed using trimmomatic [66] and aligned to Ensemble (build hg19) using bwa [67] with default settings. Duplicates reads were removed using Picard tools MarkDuplicates, and each aligned read was shifted towards the Tn5 cut site as described [38]. As reported, we found that FAST-ATAC resulted in low percentages of reads mapping to mitochondria((Supplementary Figure 4a). Regions of open chromatin were determined by combining alignment files across replicates and using MACS v.1.4.3 [68]. Open chromatin regions were merged between both high and low samples to form a universal set of peaks (peakome), and regions from ENCODE blacklist removed.

Data analysis

SEAHORSE:The data were exported and analysed using Excel V.16 (Microsoft) and Wave Software V2.4 (Agilent).
DNA METHYLATION: Reads were aligned to a bisulfite converted human genome (hg38), using Bismark [63] Methylation calls were made with bismark_methylation_extractor [63]. Analysis of methylation over CpG islands was performed using Seqmonk [64] where only CGIs with 10 or more informative CpG sites were considered. FastQC, trim_galore, and Seqmonk are all available from www.bioninformatics.babraham.ac.uk.

ATAC-SEQ: Reads were quantified for each interval in the peakome using bedtools [69] and normalized using TMM method [70]. We found that each sample had a high fraction of reads in the peakome, showed strong enrichment for open chromatin at transcription start sites, and characteristic distribution of fragments showing nucleosome free regions and mono- and di nucleosome patterns, indicating high quality ATAC libraries (Supplementary Figure b-d).

Data exploration using principle component analysis (PCA) found that the first PCA,explaining 43% of the variance, separated high and low populations, while the second PCA represented potential batch effects (Supplementary Figure 4e-f). Based on these observations, a general linearized model in edgeR [70] was used to identify differences 908 in DNA accessibility between populations including batch as a covariate. Peak set annotations and enrichments were calculated using LOLA [40] by comparing peaks more accessible in either high or low populations (FDR < 0.01, Table S1) with the universal set of DNAse hypersensitivity sites as the universe against the LOLA core. (Supplementary Table 7). Statistical analysis, data exploration, and visualization was performed using R (http://www.R-project.org).
scRNA-seq: scRNA-sequencing reads were mapped to the GRCm38 mouse genome using the Subread aligner [52] and assigned to genes using scPipe [53] with ENSEMBL v86 annotation. Gene counts were exported as a matrix by scPipe with UMI-aware counting and imported into R. Heatmaps were generated on normalised expression values using heatmap2 from the gplots package with row normalisation. Dimensionality reduction was performed on normalised log2-cpm expression values with size factors from computeSumFactors in scran [54]. We used BLAT [57] to compare every annotated human exon in ENSEMBL release 86 (737,982 unique exons across 63,305 genes) against the human (hg38) and Macaca fascicularis (macFas5) genomes. We processed all 2526 files from [30] (from 390 cells) with sickle [https://github.com/najoshi/sickle] to remove bad reads and trim low quality bases from the 3' end. We then mapped all reads to macFas5 using Rsubread 1.20.6 and R 3.2.2, allowing up to 2 mismatches and 2 indels per 50bp read, which is proportional to our setting of 5 mismatches or indels for a 100bp read. Mapped reads were assigned to the orthologous metaexon list using featureCounts at both the single metaexon and whole-gene level, and summed within individuals in R 3.2.2. Quality control of raw data was assessed using FASTQC and visualised using MultiQC. The scPipe package v1.0 for R was used to count genes based on UMI profile. Gene expression was normalised using scaterv1.6.1and scran v1.6.6packages for R. FASTQ files were aligned to hg38 using the Subread package v1.26.1 for R statistical software, aligned reads were re-annotated to exons using ENSEMBL v86 transcriptome to define the exon/intron mapping rate. PCA was performed on the merged data using prcomp function in the stats base package for R statistical software version 3.3.2. Downstream analysis of the principal components was performed using the mixOmics package version 6.3.1 for R version 3.3.2.Panther.db was used to perform Fischer's exact test for over-representation of ontological terms in gene sets of interest. Libraries were trimmed using trimmomatic [66] and aligned to Ensemble (build hg19) using bwa [67] with default settings. Duplicates reads were removed using Picard tools MarkDuplicates, and each aligned read was shifted towards the Tn5 cut site as described [38]. As reported, we found that FAST-ATAC resulted in low percentages of reads mapping to mitochondria (Supplementary Figure 4a). Regions of open chromatin were determined by combining alignment files across replicates and using MACS v.1.4.3 [68]. Open chromatin regions were merged between both high and low samples to form a universal set of peaks (peakome), and regions from ENCODE blacklist removed. Reads were quantified for each interval in the peakome using bedtools [69] and normalized using TMM method [70]. We found that each sample had a high fraction of reads in the peakome, showed strong enrichment for open chromatin at transcription start sites, and characteristic distribution of fragments showing nucleosome free regions and mono- and di-nucleosome patterns, indicating high quality ATAC libraries (Supplementary Figure b-d). Data exploration using principle component analysis (PCA) found that the first PCA, explaining 43% of the variance, separated high and low populations, while the second PCA represented potential batch effects (Supplementary Figure 4e-f). Based on these observations, a general linearized model in edgeR [70] was used to identify differences in DNA accessibility between populations including batch as a covariate. Peak set annotations and enrichments were calculated using LOLA [40] by comparing peaks more accessible in either high or low populations (FDR < 0.01, Table S1) with the universal set of DNAse hypersensitivity sites as the universe against the LOLA core. (Supplementary Table 7). Statistical analysis, data exploration, and visualization was performed using R (http://www.R-project.org).

For manuscripts utilizing custom algorithms or software that are central to the research but not yet described in published literature, software must be made available to editors/reviewers. We strongly encourage code deposition in a community repository (e.g. GitHub). See the Nature Research guidelines for submitting code & software for further information.

Data

Policy information about availability of data

All manuscripts must include a data availability statement. This statement should provide the following information, where applicable:

- Accession codes, unique identifiers, or web links for publicly available datasets
- A list of figures that have associated raw data
- A description of any restrictions on data availability

RNA-seq, scRNA-seq, and ATAC-seq data are available at Gene Expression Omnibus under accession Superseries GSE119326 (RNA-seq, GSE119324, scRNA-seq GSE119323, ATAC-seq GSE147338).

Field-specific reporting

Please select the one below that is the best fit for your research. If you are not sure, read the appropriate sections before making your selection.

☒ Life sciences ☐ Behavioural & social sciences ☐ Ecological, evolutionary & environmental sciences

For a reference copy of the document with all sections, see nature.com/documents/nr-reporting-summary-flat.pdf

Life sciences study design

All studies must disclose on these points even when the disclosure is negative.

Sample size	For quantitative studies sample sizes were chosen so as to yield standard deviations of around 10% or less based on previous results. Sample sizes were estimated to yield this degree of variability based on pilot experiments, and no calculation was performed.
Data exclusions	NONE
Replication	MINIMUM OF THREE BIOLOGICAL REPLICATES; Attempts at replication were successful. Some studies (e.g. germ cell induction or colony

Replication	formation) gave different yields, but differences between experimental groups were always consistent within experiments. Actual data points from biological replicates are provided in the manuscript or supplemental information.
Randomization	Most comparisons were between cell populations isolated by flow cytometry; there was no "assignment" to treatment groups.
Blinding	No blinding was carried out; most quantitative analyses were performed by instruments, and and where direct observations were required (colony counts, microscopy) two independent observers carried out assessment. Actual assays and micrographs are shown in the figures.

Reporting for specific materials, systems and methods

We require information from authors about some types of materials, experimental systems and methods used in many studies. Here, indicate whether each material, system or method listed is relevant to your study. If you are not sure if a list item applies to your research, read the appropriate section before selecting a response.

Materials & experimental systems

n/a	Involved in the study
☐	☒ Antibodies
☐	☒ Eukaryotic cell lines
☒	☐ Palaeontology
☒	☐ Animals and other organisms
☒	☐ Human research participants
☒	☐ Clinical data

Methods

n/a	Involved in the study
☒	☐ ChIP-seq
☐	☒ Flow cytometry
☒	☐ MRI-based neuroimaging

Antibodies

Antibodies used

Antibodies used in this study

For commercial reagents, concentration of antibodies used varied in different experiments over the course of the study, but was always within the manufacturer's suggested range for the application in question.

Flow cytometry analysis and sorting and immunostaining of hPSC

GCTM-2 mouse IgM Pera laboratory (neat hybridoma supernatant)
TG30 anti-CD9, mouse IgG2a Pera laboratory (neat hybridoma supernatant)
Both validated by immunofluorescence and flow cytometry on hPSC cell line WA09.
See also DOI: 10.1038/nbt1318

Mouse anti-human EPCAM-BV421 (EBA-1, IgG1 BD Cat # 563180)
Validation and references at: https://www.bdbiosciences.com/us/applications/research/stem-cell-research/cancer-research/mouse/bv421-mouse-anti-human-cd326-eba-1/p/563180.

Primary antibodies GCTM-2 and TG30 were detected using goat anti-mouse IgM-AF647 (A21238) and goat anti-mouse IgG2a-AF488 (A21131), respectively (Life Tech, Carlsbad, CA)
Validation and references at:
https://www.thermofisher.com/antibody/product/Goat-anti-Mouse-IgM-Heavy-Chain-Secondary-Antibody-Polyclonal/A-21238
https://www.thermofisher.com/antibody/product/Goat-anti-Mouse-IgM-Heavy-Chain-Secondary-Antibody-Polyclonal/A-21238

Rat anti-mouse IgG2a Secondary Antibody, PE/Cy7 (RMG2a-62, 407107; Biolegend) was used to detect TG30 in experiments that used FUCCI cell lines or for the Click-iT® EdU Flow Cytometry Assay
Validation and references at:
https://www.biolegend.com/en-us/products/pe-anti-mouse-igg2a-6522

GCTM-2 was visualized in immunofluorescence using goat anti-mouse IgM AlexaFluor 488 (Thermo-Fisher A21042).
Validation and references at:
https://www.thermofisher.com/antibody/product/Goat-anti-Mouse-IgM-Heavy-chain-Cross-Adsorbed-Secondary-Antibody-Polyclonal/A-21042

Differentiation into PGC-like cells

For flow cytometry:
anti-human and mouse ITGA6 BV421 (GoH3, Rat IgG2a, Biolegend 313624) and mouse anti-human CD236-PerCP (9C4, Mouse IgG2b, Biolegend 324213).
Validation and references at:
https://www.biolegend.com/en-us/products/brilliant-violet-421-anti-human-mouse-cd49f-antibody-8644
https://www.biolegend.com/en-us/products/percpcyanine55-anti-human-cd326-epcam-antibody-4252

For immunofluorescence:
rabbit antibody against PRDM1 (rabbit monoclonal IgG clone C14A4 9115 from Cell Signaling Technology) or NANOS3 (rabbit

antisera, Abcamab70001)

Validation and references at
https://www.cellsignal.com/products/primary-antibodies/blimp-1-prdi-bf1-c14a4-rabbit-mab/9115
https://www.abcam.com/nanos3-antibody-ab70001.html

Detection using goat anti-rabbit AF488 antisera (Thermofisher A32731)
Validation and references at:
https://www.thermofisher.com/antibody/product/Goat-anti-Rabbit-IgG-H-L-Highly-Cross-Adsorbed-Secondary-Antibody-Polyclonal/A32731

Tri-lineage differentiation

Rabbit anti-PAX6 polyclonal IgG (Biolegend Poly19013); goat anti-T polyclonal IgG (R&D AF2085); goat anti-SOX17polyclonal IgG (R&D AF1924)

Validation and references at:
https://www.biolegend.com/en-us/products/purified-anti-pax-6-antibody-11511
https://www.rndsystems.com/products/human-mouse-brachyury-antibody_af2085
https://www.rndsystems.com/products/human-sox17-antibody_af1924

Secondary antibodies (donkey anti-rabbit IgG Alexa Fluor Plus 488 A21206; donkey anti-goat IgG Alexa Fluor Plus 488 A11070, both from Thermofisher)

Validation and references at:
https://www.thermofisher.com/antibody/product/Donkey-anti-Rabbit-IgG-H-L-Highly-Cross-Adsorbed-Secondary-Antibody-Polyclonal/A-21206
https://www.thermofisher.com/antibody/product/Goat-anti-Rabbit-IgG-H-L-Cross-Adsorbed-Secondary-Antibody-Polyclonal/A-11070

Validation

Antibodies used in this study

For commercial reagents, concentration of antibodies used varied in different experiments over the course of the study, but was always within the manufacturer's suggested range for the application in question.

Flow cytometry analysis and sorting and immunostaining of hPSC

GCTM-2 mouse IgM Pera laboratory (neat hybridoma supernatant)
TG30 anti-CD9, mouse IgG2a Pera laboratory (neat hybridoma supernatant)
Both validated by immunofluorescence and flow cytometry on hPSC cell line WA09.
See also DOI: 10.1038/nbt1318

Mouse anti-human EPCAM-BV421 (IgG1 BD Cat # 563180)
Validation and references at: https://www.bdbiosciences.com/us/applications/research/stem-cell-research/cancer-research/mouse/bv421-mouse-anti-human-cd326-eba-1/p/563180.

Primary antibodies GCTM-2 and TG30 were detected using goat anti-mouse IgM-AF647 (A21238) and goat anti-mouse IgG2a-AF488 (A21131), respectively (Life Tech, Carlsbad, CA)
Validation and references at:
https://www.thermofisher.com/antibody/product/Goat-anti-Mouse-IgM-Heavy-Chain-Secondary-Antibody-Polyclonal/A-21238
https://www.thermofisher.com/antibody/product/Goat-anti-Mouse-IgM-Heavy-Chain-Secondary-Antibody-Polyclonal/A-21238

Rat anti-mouse IgG2a Secondary Antibody, PE/Cy7 (RMG2a-62, 407107; Biolegend) was used to detect TG30 in experiments that used FUCCI cell lines or for the Click-iT® EdU Flow Cytometry Assay
Validation and references at:
https://www.biolegend.com/en-us/products/pe-anti-mouse-igg2a-6522

GCTM-2 was visualized in immunofluorescence using goat anti-mouse IgM AlexaFluor 488 (Thermo-Fisher A21042).
Validation and references at:
https://www.thermofisher.com/antibody/product/Goat-anti-Mouse-IgM-Heavy-chain-Cross-Adsorbed-Secondary-Antibody-Polyclonal/A-21042

Differentiation into PGC-like cells

For flow cytometry:
anti-human and mouse ITGA6 BV421 (Rat IgG2a, Biolegend 313624) and mouse anti-human CD236-PerCP (Mouse IgG2b, Biolegend 324213).
Validation and references at:
https://www.biolegend.com/en-us/products/brilliant-violet-421-anti-human-mouse-cd49f-antibody-8644
https://www.biolegend.com/en-us/products/percpcyanine55-anti-human-cd326-epcam-antibody-4252

For immunofluorescence:

rabbit antibody against PRDM1 (rabbit monoclonal IgG clone 9115 from Cell Signaling Technology) or NANOS3 (rabbit antisera, Abcamab70001)

Validation and references at
https://www.cellsignal.com/products/primary-antibodies/blimp-1-prdi-bf1-c14a4-rabbit-mab/9115
https://www.abcam.com/nanos3-antibody-ab70001.html

Detection using goat anti-rabbit AF488 antisera (Thermofisher A32731)
Validation and references at:
https://www.thermofisher.com/antibody/product/Goat-anti-Rabbit-IgG-H-L-Highly-Cross-Adsorbed-Secondary-Antibody-Polyclonal/A32731

Tri-lineage differentiation

Rabbit anti-PAX6 polyclonal IgG (Biolegend Poly19013); goat anti-T polyclonal IgG (R&D AF2085); goat anti-SOX17polyclonal IgG (R&D AF1924)

Validation and references at:
https://www.biolegend.com/en-us/products/purified-anti-pax-6-antibody-11511
https://www.rndsystems.com/products/human-mouse-brachyury-antibody_af2085
https://www.rndsystems.com/products/human-sox17-antibody_af1924

Secondary antibodies (donkey anti-rabbit IgG Alexa Fluor Plus 488 A21206; donkey anti-goat IgG Alexa Fluor Plus 488 A11070, both from Thermofisher)

Validation and references at:
https://www.thermofisher.com/antibody/product/Donkey-anti-Rabbit-IgG-H-L-Highly-Cross-Adsorbed-Secondary-Antibody-Polyclonal/A-21206
https://www.thermofisher.com/antibody/product/Goat-anti-Rabbit-IgG-H-L-Cross-Adsorbed-Secondary-Antibody-Polyclonal/A-11070

Eukaryotic cell lines

Policy information about cell lines

Cell line source(s): Human embryonic stem cells (WA09 and FUCCI-G1). WA09 was obtained from WiCell. The FUCCI cells, a derivative of WA09, were a gift from Prof Jonathan S. Draper [29].

Authentication: Cell lines were authenticated by the provider using STR profiling, Master banks were established and cell lines were used within 10-15 passages after establishment.

Mycoplasma contamination: Cells were routinely tested for mycoplasma on a monthly basis and found to be negative

Commonly misidentified lines (See ICLAC register): No commonly misidentified cell lines were used in this study.

Flow Cytometry

Plots

Confirm that:

☒ The axis labels state the marker and fluorochrome used (e.g. CD4-FITC).

☒ The axis scales are clearly visible. Include numbers along axes only for bottom left plot of group (a 'group' is an analysis of identical markers).

☒ All plots are contour plots with outliers or pseudocolor plots.

☒ A numerical value for number of cells or percentage (with statistics) is provided.

Methodology

Sample preparation: WA09 cells grown for 24 hours in the presence of Y-27632 Rho kinase inhibitor were dissociated using Accutase, harvested, and examined under the microscope to ensure that most of the cells had not completely dissociated into single cells. Immunolabelling was carried out by incubation in a primary antibody cocktail containing TG30 anti-CD9 antibody and GCTM-2 for 20 min at 4° C, followed by incubation in a secondary antibody cocktail containing goat anti-mouse IgG2a AlexaFluor 488 antibody and goat anti-mouse IgM AlexaFluor 647 antibody, diluted in 2% FBS in DMEM-F12 flow cytometry buffer, again at 4° C for 20 min. The cells were resuspended the cells in 1x DAPI in cold mTeSR1before filtering the suspension through a 35 µm cell strainer to remove any larger aggregates and debris.

Instrument: BD FACSAria III

Software	FCS Express or FLO JO
Cell population abundance	Cell population abundances were as follows: Double stained cells (GCTM2 and TG30) were sorted into several populations: GCTM-2lowCD9low, GCTM-2highCD9high or GCTM-2highCD9highEPCAMhigh. The low population consists of cells with low (bottom 25%) expression of GCTM2 and TG30, the GCTM-2highCD9high subset consists of the top 25% of cells expressing GCTM2 and TG30 whereas the GCTM-2highCD9highEPCAMhigh subset is a fraction of the GCTM-2highCD9high population with the highest expression of EPCAM, representing ~10% of GCTM-2highCD9high fraction.
Gating strategy	Unstained controls were used to determine patterns of forward and side scatter and to gate out debris and doublets<br>Viability was determined with Ghost Dye, Propidium Iodide, or 7-AAD.<br>Single stained cells were used in compensation, and cells stained with secondary antibodies only were used to establish negative cutoffs for positive staining.<br>Exemplary gating strategies are illustrated in Supplementary Figure 12.

☒ Tick this box to confirm that a figure exemplifying the gating strategy is provided in the Supplementary Information.
