# Supplementary material for: Unique properties of a subset of human pluripotent stem cells with high capacity for self-renewal
Source: Nat Commun. 2020 May 15;11:2420. doi: 10.1038/s41467-020-16214-8 (PMC7229198; doi:10.1038/s41467-020-16214-8)
Supplement: Supplementary file 4 — Description of Additional Supplementary Files [file 41467_2020_16214_MOESM4_ESM.pdf]

Description of Additional Supplementary Files

Supplementary Movie 1:
Fate of aggregates of GCTM-2highCD9high cells for 1-24 hours post-plating

Supplementary Movie 2:
Fate of aggregates of GCTM-2midCD9mid cells for 1-24 hours post-plating.

Supplementary Data 1.
Differential chromatin accessible peaks between populations.

Supplementary Data 2.
Results from locus overlap enrichment analysis (LOLA) for chromatin accessible peaks from GCTM-2highCD9highEPCAMhigh and GCTM-2midCD9mid populations.

Supplementary Data 3.
RNA-seq analysis of differential gene expression in GCTM-2highCD9highEPCAMhigh subpopulation and unsorted cells (general population).
